# Forelimb Motor Learning and Memory Consolidation Drives Distinct Oligodendrocyte Plasticity to Regulate Task‐related Neuronal Activity

**DOI:** 10.1002/advs.202505367

**Published:** 2025-09-30

**Authors:** Shuming Wang, Nuo Xu, Wenwen Wang, Yongxiang He, Yuqian Yang, Liuning Zhang, Yanping Zou, Yuehua He, Huiliang Li, Liang Gao, Lin Xiao

**Affiliations:** ^1^ Key Laboratory of Brain Cognition and Education Sciences of Ministry of Education Institute for Brain Research and Rehabilitation Guangdong Key Laboratory of Mental Health and Cognitive Science and Center for Studies of Psychological Application South China Normal University Guangzhou 510631 China; ^2^ Wolfson Institute for Biomedical Research University College London Gower Street London WC1E 6BT UK; ^3^ Guangdong Provincial Key Laboratory of Brain Connectome and Behavior CAS Key Laboratory of Brain Connectome and Manipulation The Brain Cognition and Brain Disease Institute (BCBDI) Shenzhen Institutes of Advanced Technology Chinese Academy of Sciences Shenzhen‐Hong Kong Institute of Brain Science‐Shenzhen Fundamental Research Institutions Shenzhen 518055 China

**Keywords:** adaptive OL/myelin plasticity, calcium activity, memory consolidation, motor learning, single pellet reaching task

## Abstract

Motor learning induces oligodendrocyte (OL) dynamics/plasticity during learning. However, it remains unclear whether different adaptive OL dynamics are required for different phases of motor learning and how they regulate neuronal activity. Here, we showed reduced oligodendrogenesis accompanied by elongated node length in the contra‐rostral forelimb area (cRFA) motor cortex during learning of the forelimb reaching task, both of which correlate with the learning performance. However, we observed increased oligodendrogenesis during the motor memory consolidation phase, which also correlates with the motor skill maintenance. Strikingly, *Myrf* conditional knockout (OPC‐*Myrf*‐cKO) mice, in which oligodendrogenesis can be artificially blocked, showed improved learning performance along with increased node length and increased task‐related neuronal activity in the cRFA when *Myrf* deletion (i.e., oligodendrogenesis blockade) is introduced prior to learning. However, they showed impaired rehearsal performance accompanied by decreased task‐related neuronal activity when gene deletion is induced after learning. These findings suggest that motor learning and consolidation may drive distinct OL plasticity to fine‐tune task‐related neuronal activity required at different phases.

## Graphical abstract

Genetic and chemical tracking of oligodendrogenesis, combining fiber photometric neuronal activity recording, reveals that distinct oligodendrocyte plasticities are adopted during different phases of motor learning to fine‐tune task‐related neuronal activity, with a preferential involvement of oligodendrogenesis suppression and node lengthening (type 2 oligodendrocyte plasticity) during learning and oligodendrogenesis enhancement (type 1 oligodendrocyte plasticity) during consolidation in a single pellet reaching task.

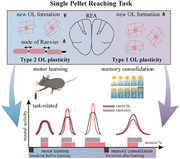

## 1 Introduction

In the central nervous system (CNS), oligodendrocytes (OLs) extend numerous myelin sheaths to enwrap axons, thereby accelerating action potential conduction, providing metabolic support, and maintaining axonal integrity.^[^ ^1^, ^2^, ^3^ ^]^ Myelination develops in a similar way in humans and mice, with basic myelination completed in the early postnatal period (first 6 weeks in mice and adolescence in humans), followed by subtle changes with life experience, and demyelination in the ageing phase.^[^ ^4^, ^5^, ^6^, ^7^ ^]^ As new OL and myelin are continuously produced in the brain throughout life,^[^ ^8^ ^]^ a novel form of brain plasticity involving this change, i.e., adaptive OL/myelin plasticity, has been proposed and suggested to play a crucial role in various brain functions, including learning and memory.^[^ ^9^, ^10^, ^11^, ^12^ ^]^

In fact, neuronal activity and experience‐induced OL/myelin plasticity in adulthood can either be the formation of new myelin by newly generated OLs (type 1 OL/myelin plasticity) or the remodeling of pre‐existing myelin sheaths by old OLs (type 2 OL/myelin plasticity).^[^ ^10^, ^12^, ^13^, ^14^, ^15^, ^16^ ^]^ Myelin regulatory factor (*Myrf*) encodes a transcription factor that is necessary for OL differentiation and survival.^[^ ^17^ ^]^ Conditional knockout (cKO) of *Myrf* in OL precursor cells (OPCs) can block the production of new OL/myelin, which has been a well‐used approach to investigate the role of type 1 OL/myelin plasticity in various brain functions.^[^ ^18^, ^19^ ^]^ For example, McKenzie et al.^[^ ^18^ ^]^ and Xiao et al.^[^ ^19^ ^]^ have shown that oligodendrogenesis is required for a “complex wheel” motor task from the onset of learning, and *Pdgfrα‐CreER^T2^: Myrf* ^loxP/loxP^ mice show impairment during motor learning. In addition, these mice also exhibit deficits in the T‐maze and radial‐arm‐maze task, suggesting the involvement of new OL and myelin production in spatial working memory.^[^ ^20^ ^]^ Using a similar *Myrf*‐cKO approach, Pan et al.^[^ ^21^ ^]^ and Steadman et al.^[^ ^22^ ^]^ show that water maze learning and memory consolidation, as well as the long‐term memory formation and remote rehearsal of contextual fear, require oligodendrogenesis, whereas fear learning and its short‐term rehearsal are unaffected. Collectively, these studies suggest the pattern of adaptive OL production and myelination that is required to modify and reinforce task‐related neural circuits may be context‐ and phase‐dependent.

Interestingly, repetitive transcranial magnetic stimulation and the radial arm maze spatial learning task have been shown to alter the length of the nodes of Ranvier and the size of the periaxonal space within active brain regions, i.e., to modulate pre‐existing myelin plasticity independently of oligodendrogenesis.^[^ ^23^ ^]^ Evidence for the involvement of pre‐existing myelin remodeling, i.e., type 2 OL/myelin plasticity in learning and memory, also comes from direct in vivo two‐photon imaging. For example, in a single pellet reaching task (SPRT), Bacmeister et al.^[^ ^24^, ^25^ ^]^ shows that in the learning phase, motor training remodels the pre‐existing myelin sheath of learning‐activated axons by increasing their retraction, which result in elongation of the nodes of Ranvier, concomitant of a transiently suppressed oligodendrogenesis. However, within the following 2 weeks post‐learning, OPC differentiation, OL generation and myelin sheath remodeling are all increased. Whereas in another study, using the same imaging technique, Hughes et al.^[^ ^13^ ^]^ shows that sensory enrichment increases oligodendrogenesis but does not remodel the pre‐existing myelin sheaths. Thus, type 2 OL/myelin plasticity also appears to be context‐ and phase‐dependent.

Motor learning engages a complex network of brain regions, including motor cortex, white matter (WM), striatum, motor thalamus (MT), cerebellum (CBM), brainstem, and basal ganglia (BG).^[^ ^26^, ^27^ ^]^ For dexterous forelimb motor tasks, motor cortex specifies rostral forelimb area (RFA) and the caudal forelimb area (CFA),^[^ ^28^, ^29^, ^30^, ^31^ ^]^ with RFA evoking limb grasping and CFA evoking tapping. An additional grasping representation area, the lateral forelimb area has also been identified.^[^ ^28^ ^]^ There are several circuits that send inputs to the primary motor cortex (M1), e.g. MT receives sensory messages from BG and sends integrated information to M1, which sends commands to perform tasks.^[^ ^27^, ^32^, ^33^, ^34^, ^35^, ^36^ ^]^ Cortical‐cortical (CC) connection between motor forelimb areas is also important for modulating downstream information.^[^ ^37^ ^]^ Numerous studies have demonstrated the critical role of neuronal/synaptic plasticity during motor learning and memory.^[^ ^38^, ^39^, ^40^, ^41^, ^42^ ^]^ While in the SPRT task, highly specific synaptic plasticity is identified during the learning and formation of long‐lasting motor memory traces in the corticostriatal circuit.^[^ ^38^ ^]^

Neuronal/synaptic plasticity can regulate neural information processing, but it remains largely unknown whether and how OL/myelin plasticity would have similar functions. In a transgenic model of myelin impairment, mice unable to learn a self‐initiated lever‐pull task show deficits in task‐related calcium transients and thalamocortical axonal conduction and synchronization, suggesting that normal myelination and OL genesis are required for learning‐related neural activity.^[^ ^43^ ^]^ Both the Hughes^[^ ^25^ ^]^ and Young^[^ ^23^ ^]^ groups provide evidence that changes in the nodes of Ranvier lead to changes in conduction velocity, but how these changes optimize the encoding of movement commands from the motor cortex is unclear. In particular, while the long‐term (up to 3 months) in vivo imaging of motor cortex layers I‐III, focusing on a defined field of motor cortex, it remains to be seen whether this would be the case in a global view of the forelimb motor cortex. Furthermore, the role of the bidirectional changes in OL/myelin dynamics in task‐related neural activity in these two periods remains to be elucidated.

In this study, using both genetic and chemical labelling and tracking of OPC proliferation and differentiation, we systematically mapped OL dynamic profiles across several different brain regions after SPRT learning. We observed a decrease in oligodendrogenesis, accompanied by an increase in the length of the nodes of Ranvier in the contralateral RFA (cRFA) of learners. Interestingly, the increase in nodal length correlated with the degree of increase in oligodendrogenesis, and they both correlate with the motor learning performance, suggesting a causal relationship between nodal lengthening and skill acquisition. Strikingly, *Myrf*‐cKO mice, in which OL production was genetically suppressed showed improved learning ability and increased nodal length in the cRFA than control mice when *Myrf* deletion in OPC (i.e., oligodendrogenesis blockade) was induced prior to the reaching task. What's more, their behavioral improvement was accompanied by higher movement‐related neuronal activity during reaching and more cFos‐positive neurons in the cRFA, as well as lower variance in reaching. We further investigated the changes in oligodendrogenesis during the memory consolidation phase and found a significant and persistent increase in both OPC proliferation and new OL production in the cRFA of learners, which correlates with the skill rehearsal performance. Notably, blockade of new OL/myelin production in *Myrf*‐cKO mice after motor learning impaired their skill rehearsal and task‐related neuronal activation, suggesting that prolonged post‐training oligodendrogenesis is required for motor memory consolidation. Taken together, our results suggest that distinct OL dynamics/plasticity are adopted by forelimb motor learning during different phases to fine‐tune task‐related neuronal activity, with a preferential involvement of oligodendrogenesis suppression and node lengthening (type 2 OL plasticity) during learning and oligodendrogenesis enhancement (type 1 OL plasticity) during consolidation in SPRT.

## 2 Results

### 2.1 SPRT Learning Suppresses Oligodendrogenesis

**Figure 1:**
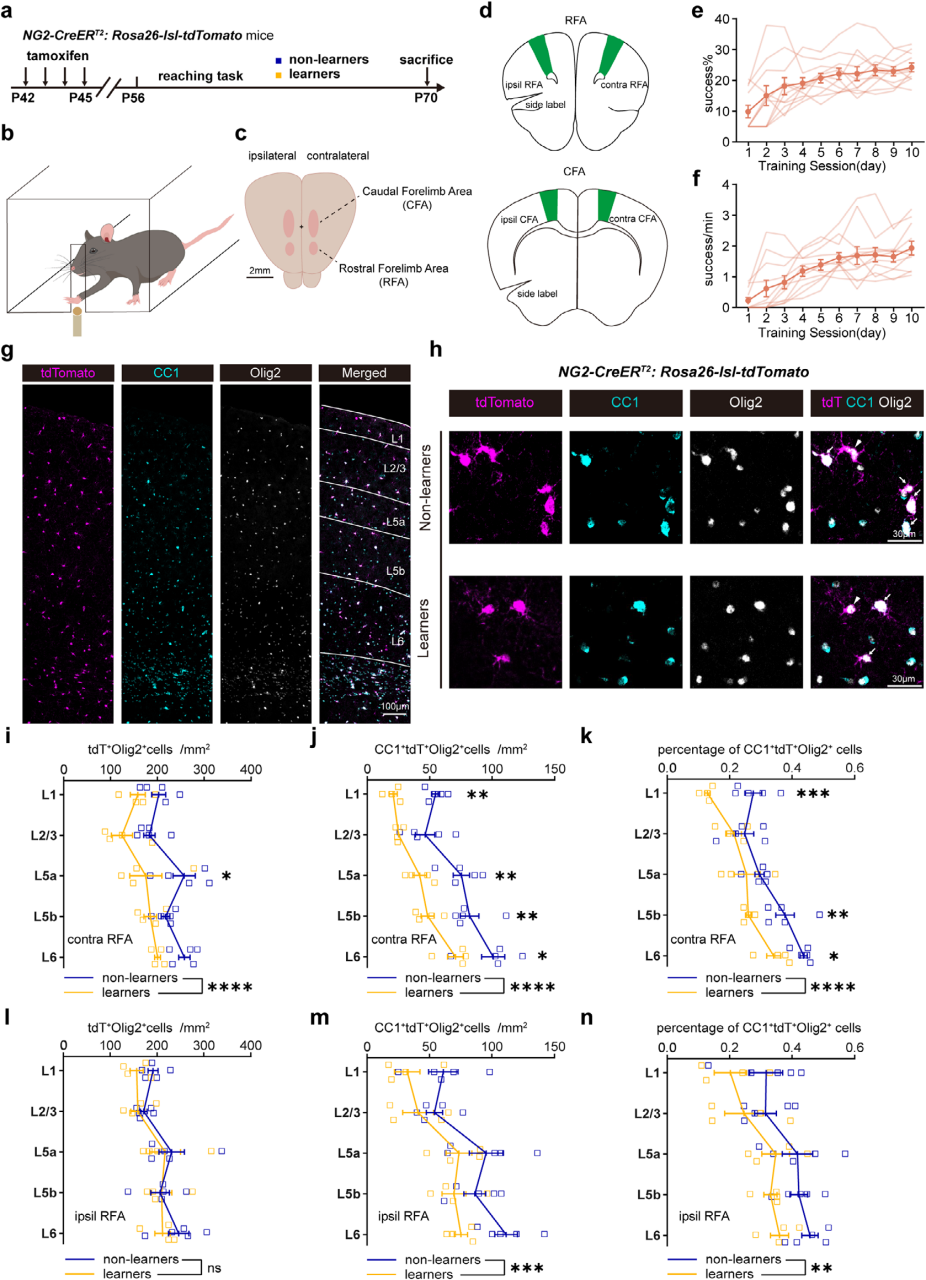
SPRT learning suppresses oligodendrogenesis in cRFA revealed in NG2‐tdT mice. A) Experimental timeline to trace OPC dynamics during reaching task. b,c) Schematic drawings of the forelimb reaching task in a right‐handed mouse (b) and corresponding brain tissue (c). d) Schematic drawings of coronal brain slices, including RFA (top) and CFA (bottom). e,f) Behavior performance of mice during a 10‐day learning session. Thin lines represent individual mice and the bold represents the average. n = 11 mice. Error bar, s.e.m. Repeated‐measured one‐way ANOVA: F (2.400, 24.00) = 8.528, p = 0.0010 (e), F (2.168, 21.68) = 17.72, p < 0.0001 (f). g) Representative motor cortex image of tdTomato (magenta), CC1 (cyan), and Olig2 (grey) immunostaining and layers identification in motor cortex. H) Representative high magnification image of cell co‐localization from non‐learners and learners in *NG2‐CreER^T2^: Rosa26‐lsl‐tdTomato* transgenic mice. Arrows denote tdT^+^CC1^+^Olig2^+^ cells, arrowheads denote tdT^+^CC1^−^Olig2^+^ cells. i–k). Cell number density of tdT^+^Olig2^+^, CC1^+^ tdT^+^Olig2^+^, and percentage of CC1^+^ tdT^+^Olig2^+^ cells in cRFA of learners (orange, n = 4 mice per layer) and non‐learners (blue, n = 5 mice per layer). Two‐way ANOVA with Šídák's post hoc test: i, non‐learners versus learners (training factor), F (1, 35) = 23.14, p < 0.0001; in L5a, adjusted p = 0.0143; j, training factor: F (1, 35) = 50.31, p < 0.0001; in L1, adjusted p = 0.0093, in L5a, adjusted p = 0.0013, in L5b, adjusted p = 0.0103, in L6, adjusted p = 0.0251; k), training factor: F (1, 35) = 32.73, p < 0.0001; in L1, adjusted p = 0.0006, in L5b, adjusted p = 0.0075, in L6, adjusted p = 0.0396. Data are presented as mean ± s.e.m., * p < 0.05, ** p < 0.01, *** p <0.001, **** p < 0.0001. l‐n Cell number density of tdT^+^Olig2^+^, CC1^+^ tdT^+^Olig2^+^ and percentage of CC1^+^ tdT^+^Olig2^+^ cells in iRFA of learners (orange, n = 4 mice per layer) and non‐learners (blue, n = 5 mice per layer). Two‐way ANOVA with Šídák's post hoc test: m, non‐learners versus learners (training factor), F (1, 35) = 2.129, p = 0.1534; n, training factor: F (1, 35) = 12.90, p = 0.0010; o, training factor: F (1, 35) = 10.92, p = 0.0022. Data are presented as mean ± s.e.m., * p < 0.05, ** p < 0.01, *** p <0.001, **** p < 0.0001.

Using *NG2‐CreER^T2^: Rosa26‐lsl‐tdTomato* (NG2‐tdT) transgenic reporter mice to label and track OPCs and their newly formed OLs offspring, we systematically mapped the changes in oligodendrogenesis in the RFA, CFA, and other motor‐related brain regions in response to SPRT motor learning (**Figure** **1**a–d). During the learning session, the mice showed increased success rates and success trials per minute (Figure 1e,f). Mice of “learners” (achieved a peak success rate of over 20% on at least one day during the 10‐day training period) and “non‐learners” (never achieved a peak success rate of 20% during the entire training) were grouped as described in the Methods section (see also Figure S1a–d, Supporting Information). Both the left‐ and right‐handed learners finished their learning and achieved at least 20% success rate without detectable differences (see also Figure S1e–g, Supporting Information).

By immunolabeling of NG2 (OPC marker), CC1 (differentiated OL marker), and Olig2 (common OL lineage marker) together with tdTomato (tdT), we identified OPCs (NG2^+^tdT^+^Olig2^+^) and newly differentiated OLs (CC1^+^tdT^+^Olig2^+^) (Figure 1g,h; Figure S2a, Supporting Information) to trace the OPC cell dynamics across several main motor‐related brain regions. Given the possible heterogeneity of OPCs among different brain regions,^[^ ^44^ ^]^ to discern the possible differences in the inherent properties of OPC dynamic between different layers of the cortical grey matter, we separately counted the labeled OL lineage cells in different layers of the motor cortex from layer 1 (L1) to layer 6 (L6) for the analyses (Figure 1g). We found that over the SPRT, the density of tdT^+^Olig2^+^, CC1^+^tdT^+^Olig2^+^ cells, as well as the percentages of CC1^+^tdT^+^Olig2^+^ cells among the total tdT^+^Olig2^+^ cells, decreased significantly in multiple layers (e.g., L1, L5, L6) of the contralateral RFA (cRFA) in learners compared to non‐learners (Figure 1i–k), but not in any specific layers of the ipsilateral RFAs (iRFA) (Figure 1l–n). Moreover, the number of NG2^+^tdT^+^Olig2^+^ cells of the OPC pool also remained unaffected in both the cRFA and the iRFA (Figure S2a—c, Supporting Information). These data suggest that motor learning induces the suppression of oligodendrogenesis specifically in the cRFA. Notably, there were dramatically more CC1^+^tdT^+^Olig2^+^ cells in the deeper layer (e.g., L5, L6) than upper layer (e.g., L2/3), both in the cRFA and iRFA, in both learner and non‐learner mice (Figure 1j,k,m,n), indicating a layer‐dependent heterogeneity of OPC differentiation ability in the grey matter.

**Figure 2:**
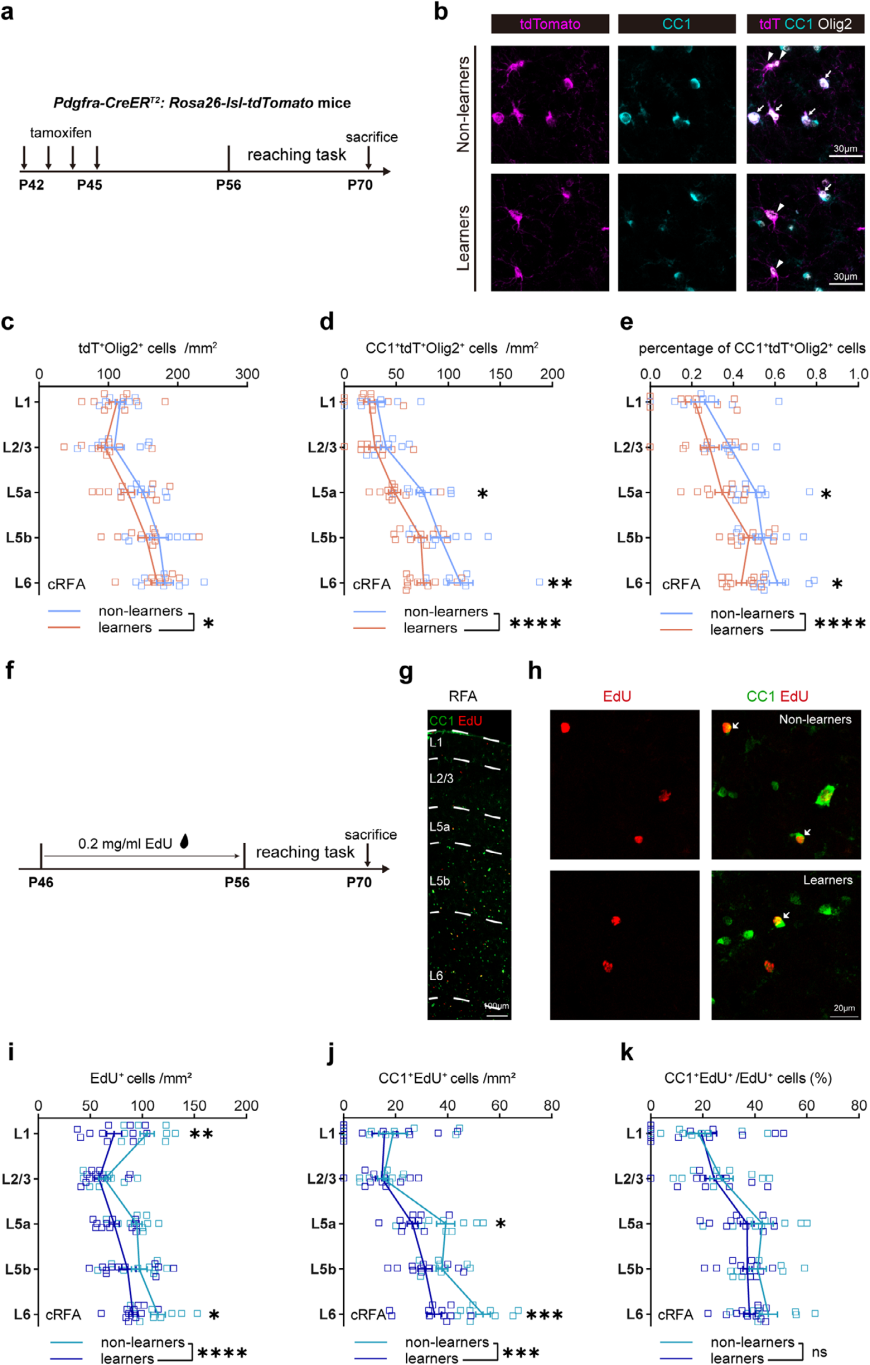
SPRT learning suppresses oligodendrogenesis in cRFA revealed in P‐tdT mice and by EdU labeling. A) Experimental timeline to trace OPC dynamics during reaching task. B) Representative high magnification image of cell co‐localization from non‐learners and learners in *Pdgfrα‐CreER^T2^: Rosa26‐lsl‐tdTomato* transgenic mice. Arrows denote tdT^+^CC1^+^Olig2^+^ cells, arrowheads denote tdT^+^CC1^−^Olig2^+^ cells. C–e) Cell number density of tdT^+^Olig2^+^, CC1^+^ tdT^+^Olig2^+^ and percentage of CC1^+^ tdT^+^Olig2^+^ cells in cRFA of learners (orange, n = 10 mice per layer) and non‐learners (light blue, n = 8 mice per layer). Two‐way ANOVA with Šídák's post hoc test: c, non‐learners versus learners (training factor), F (1, 80) = 4.041, p = 0.0478. d, training factor: F (1, 80) = 18.79, p < 0.0001; in L5a, adjusted p = 0.0411, in L6, adjusted p = 0.0047; e, training factor: F (1, 80) = 16.98, p < 0.0001, in L5a, adjusted p = 0.0355, in L6, adjusted p = 0.0232. Data are presented as mean ± s.e.m., * p < 0.05, ** p < 0.01, *** p <0.001, **** p < 0.0001. f) Experimental timeline to trace OPC dynamics during reaching task. g,h) Representative staining image with EdU (red) and CC1 (green). Arrows denote EdU^+^CC1^+^ cells. I–k) Cell number density of EdU^+^, CC1^+^EdU^+^, and percentage of CC1^+^EdU^+^ cells in cRFA of learners (blue, n = 11 mice per layer) and non‐learners (light blue, n = 9 mice per layer). Two‐way ANOVA with Šídák's post hoc test: i, non‐learners versus learners (training factor), F (1, 90) = 21.81, p < 0.0001; in L1, adjusted p = 0.0033, in L6, adjusted p = 0.0427. j, training factor: F (1, 90) = 12.97, p = 0.0005; in L5a, adjusted p = 0.0157, in L6, adjusted p = 0.0005; k, training factor: F (1, 90) = 2.464, p = 0.1200. Data are presented as mean ± s.e.m. * p < 0.05, ** p < 0.01, *** p <0.001, **** p < 0.0001.

For a double check in another reporter mouse line, we further tested *Pdgfrα‐CreER^T2^: Rosa26‐lsl‐tdTomato* (P‐tdT) transgenic mice using the same labeling strategy (**Figure** **2**a). Similarly, the density of tdT^+^Olig2^+^ and CC1^+^tdT^+^Olig2^+^ cells, as well as the percentages of CC1^+^tdT^+^Olig2^+^ cells among the total tdT^+^Olig2^+^ cells were all decreased in the cRFA (Figure 2b–e), but not in the iRFA (Figure S2d–f, Supporting Information) of learners compared to non‐learners. To obtain a triple confirmation of the decrease in oligodendrogenesis using an alternative way, we applied a well‐used chemical tracing approach by administering the thymdine analogue 5‐ethynyl‐2′‐deoxyuridine (EdU) incorporation strategy (Figure 2f).^[^ ^19^ ^]^ Consistently, there was a decrease in the number of total EdU^+^ cells and CC1^+^EdU^+^ cells, as well as the proportion of CC1^+^EdU^+^ cells among all EdU^+^ cells in the cRFA of learner mice (Figure 2g–k), but again, not in the iRFA (Figure S2g–I, Supporting Information). Taken together, we conclude that SPRT motor learning suppresses new OL production, specifically in the cRFA, probably by inhibiting both OPC proliferation and differentiation.

In addition, we examined the OL lineage cell generation in the CFA and its subcortical white matter, as well as corpus callosum (CC), and motor thalamus (MT).^[^ ^26^ ^]^ We showed that the densities of tdT^+^Olig2^+^ and CC1^+^tdT^+^Olig2^+^ cells in all these motor‐related areas remained similar between learners and non‐learners, regardless of the contralateral or ipsilateral side of the trained limb (Figures S3 and S4, Supporting Information), suggesting an exclusive sensitivity of the changes in oligodendrogenesis in the RFA but not in other motor‐related regions during reaching and grasping tasks.

### 2.2 Correlation of OL Dynamic With Motor Learning Performance

**Figure 3:**
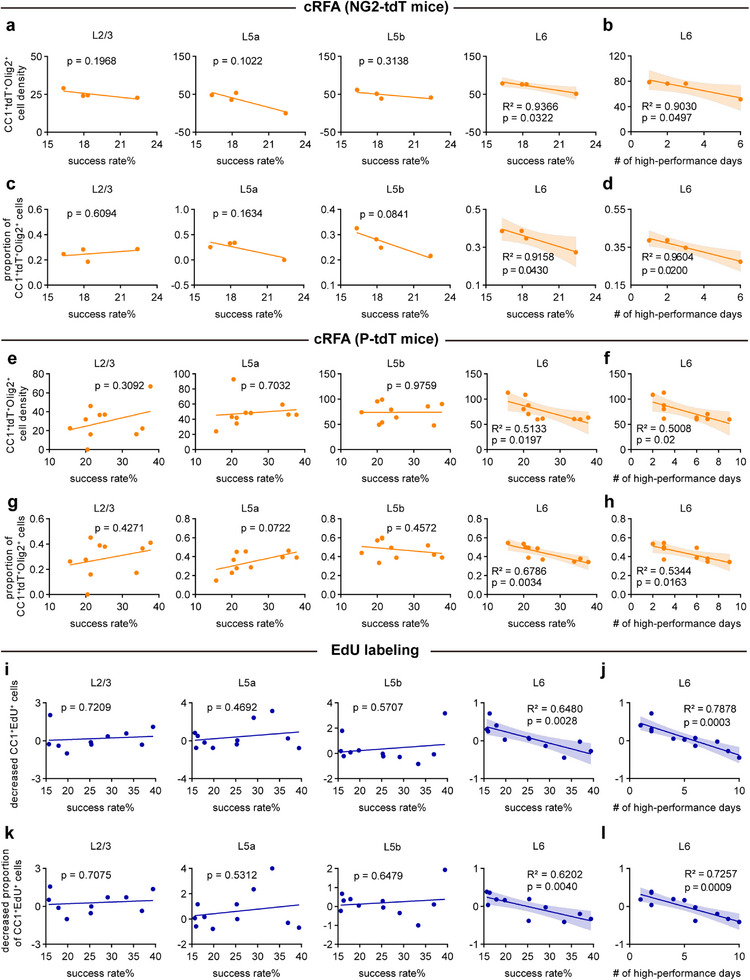
The correlation between OL dynamics and motor learning performance. a) Correlation of CC1^+^tdT^+^Olig2^+^ cell density in cRFA with reaching performance (success rate) for individual NG2‐tdT mice (n = 4 learners). B) Correlation of CC1^+^tdT^+^Olig2^+^ cell density in L6 with the number of high‐performance days over 20% success rate for individual NG2‐tdT mice (n = 4 learners). C) Correlation of the percentage of CC1^+^tdT^+^Olig2^+^ cells in cRFA with reaching performance (success rate) for individual NG2‐tdT mice (n = 4 learners). D) Correlation of the percentage of CC1^+^tdT^+^Olig2^+^ cells in L6 with the number of high‐performance days over 20% success rate for individual NG2‐tdT mice (n = 4 learners). e Correlation of CC1^+^tdT^+^Olig2^+^ cell density in cRFA with reaching performance (success rate) for individual P‐tdT mice (n = 10 learners). F) Correlation of CC1^+^tdT^+^Olig2^+^ cell density in L6 with the number of high‐performance days over 20% success rate for individual P‐tdT mice (n = 10 learners). G) Correlation of the percentage of CC1^+^tdT^+^Olig2^+^ cells in cRFA with reaching performance (success rate) for individual P‐tdT mice (n = 10 learners). H) Correlation of the percentage of CC1^+^tdT^+^Olig2^+^ cells in L6 with the number of high‐performance days over 20% success rate for individual P‐tdT mice (n = 10 learners). I) Correlation of decreased fractions of CC1^+^EdU^+^ cells relative to ipsil‐RFA with reaching performance (success rate) for individual EdU labeling mice (n = 11 learners). J) Correlation of decreased fractions of CC1^+^EdU^+^ cells relative to ipsil‐RFA with the number of high‐performance days over 20% success rate for individual EdU labeling mice (n = 11 learners). K) Correlation of decreased percentage of CC1^+^EdU^+^ cells relative to ipsil‐RFA with reaching performance (success rate) for individual EdU labeling mice (n = 11 learners). L) Correlation of decreased percentage of CC1^+^EdU^+^ cells relative to ipsil‐RFA with the number of high‐performance days over 20% success rate for individual EdU labeling mice (n = 11 learners). Lines represent linear regression, shaded area represents 95% confidence intervals (CI). Pearson correlation analysis. * p < 0.05, ** p < 0.01.

OPC proliferation and differentiation have been shown to dictate performance outcomes of working memory training.^[^ ^45^ ^]^ To investigate whether the extent of OL dynamic/plasticity is linked to the acquisition of reaching skills, we plotted the success rate of individual NG2‐tdT mice in learner group in the last training session against the density of CC1^+^tdT^+^Olig2^+^ cells and the proportion of CC1^+^tdT^+^Olig2^+^ cells (both for OPC differentiation) in both the contralateral and ipsilateral RFA. We found that across the cortical layers in the cRFA, CC1^+^tdT^+^Olig2^+^ cells and the proportion of CC1^+^tdT^+^Olig2^+^ cells in L6 were both negatively correlated with the performance of each mouse, whereas the correlations across other layers (L2/3‐L5b) were not significant (**Figure** **3**a–d). The correlations were not obvious for iRFA across the different layers, which again suggests that changes in the cRFA were driven by the dominant limb (Figure S5a,b, Supporting Information). We also plotted the success rate of individual NG2‐tdT mice against the density of tdT^+^Olig2^+^ cells in contralateral and ipsilateral RFA across different layers. We found no significant correlations between any of these factors (Figure S5c,d, Supporting Information). We then analyzed the correlation of CC1^+^tdT^+^Olig2^+^ cell density and its proportion to tdT^+^Olig2^+^ cell density with success rate and the number of high‐performance days in P‐tdT mice, we found a linear relationship in L6 of cRFA, but not in L2‐L5 (Figure 3e–h) or in any layer of iRFA (Figure S6a,b, Supporting Information). The correlation analysis to tdT^+^Olig2^+^ cell density also showed no significance in either cRFA or iRFA (Figure S6c,d, Supporting Information). We further compared the success rate of EdU labeling mice in learner group with the reduction of CC1^+^EdU^+^ cells and the percentage of CC1^+^EdU^+^ cells among EdU^+^ cells (for OPC differentiation), as well as the total reduction of EdU^+^ cells, by normalizing the corresponding data of the ipsilateral side. Consistently, the degree of reduction in CC1^+^EdU^+^ cells, as well as the percentage of CC1^+^EdU^+^ cells, displayed a linear relationship with the success rate in L6, but not in L2/3‐L5b (Figure 3i–l). However, data for the EdU^+^ cell density did not correlate with skill performance (Figure S7, Supporting Information). These data demonstrate a specific linear correlation between OL dynamics in the L6 of the cRFA, i.e., the suppression of new OL production, particularly through OPC differentiation inhibition, and the proficiency of motor performance, indicating a causal effect of oligodendrogenesis suppression on skill acquisition during the training phase of motor learning.

### 2.3 SPRT Learning Induces Node Lengthening

**Figure 4:**
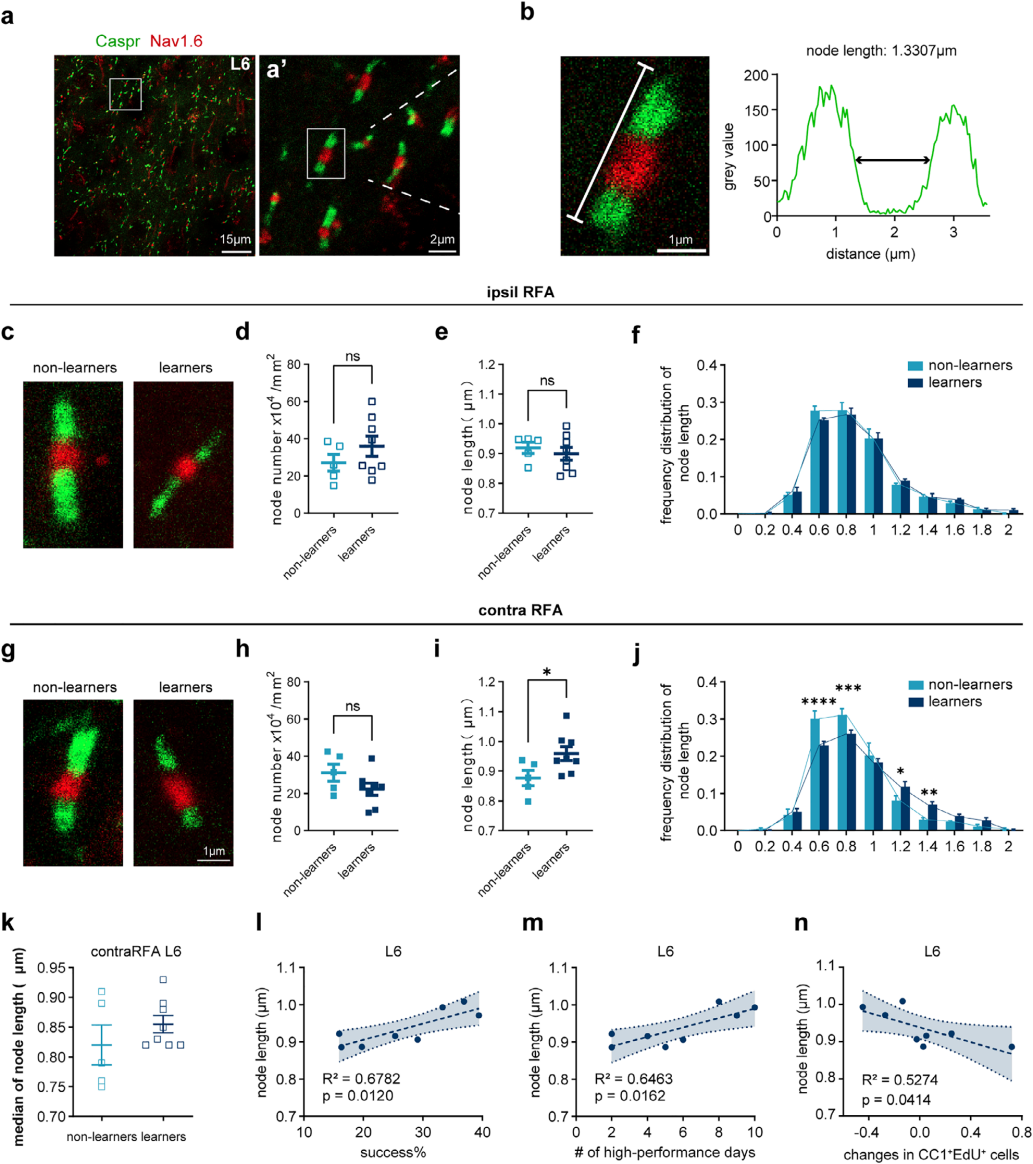
Nodal dynamics and their correlation with motor learning performance. A) Immunostaining of Caspr (green, paranode marker) and Nav1.6 (red, node marker) in L6 of RFA. Enlarged view of white square region is displayed at right (a’). b) Identification of a complete node structure and length analysis result. The length is calculated as the width of half the maximum grey value. C) Representative images of non‐learners and learners in iRFA. d‐f Node density d), node length e), and frequency distribution of node length in 0.2 µm‐bin f) in iRFA of learners (blue, n = 8 mice) and non‐learners (light blue, n = 5 mice), data are presented as mean ± s.e.m. Unpaired two‐tailed t test: d, t = 1.142, df = 11, p = 0.2779; e, t = 0.6188, df = 11, p = 0.5486. f, Scheirer‐Ray‐Hare test: F (learning factor) = 0.009, p = 0.924, F (learning factor × node length, interaction effect) = 0.565, p = 0.839. g) Representative images of non‐learners and learners in cRFA. h,i) Node density (h) and node length (i) in cRFA of learners (blue, n = 8 mice) and non‐learners (light blue, n = 5 mice), data are presented as mean ± s.e.m. Unpaired two‐tailed t test: h, t = 1.618, df = 11, p = 0.1339; i, t = 2.280, df = 11, p = 0.0436. j) Frequency distribution of node length in 0.2 µm‐bin in cRFA, each dot represents per mice, n = 5 or 8 mice per group. Data are presented as mean ± s.e.m., lines are frequency distribution curves. Scheirer‐Ray‐Hare test: F (learning factor) = 0.293, p = 0.589, F (learning factor × node length, interaction effect) = 4.951, p < 0.0001. post hoc Bonferroni's test: at 0.6‐µm length, adjusted p < 0.0001, at 0.8‐µm length, adjusted p = 0.0099, at 1.2‐µm length, adjusted p = 0.015, at 1.4‐µm length, adjusted p = 0.0011. k Median of distribution curves (j). n = 5 or 8 mice per group. Data are presented as mean ± s.e.m. Two‐tailed Mann Whitney test: p = 0.3730. l Correlation between node length in L6 of cRFA and success rate in the last training day of individual mice. Pearson correlation analysis. m Correlation between node length in L6 of cRFA and the number of high‐performance days of individual mice. Pearson correlation analysis. n Correlation between node length and decreased fraction of CC1^+^EdU^+^ cells of individual mice in L6 of cRFA. Pearson correlation analysis. ns, no significance, p > 0.05, * p < 0.05, ** p < 0.01, *** p <0.001, **** p < 0.0001.

The suppression of OL production and its linear relationship with skill performance in SPRT learning is quite surprising, as it seems to contradict previous findings by Richardson's group showing that learning another motor skill of running a “complex wheel” stimulates new OL production in both the motor cortex and CC.^[^ ^18^, ^19^ ^]^ However, our findings are consistent with the suppression of OL genesis observed using longitudinal in vivo imaging in the same SPRT learning model, accompanied by pre‐existing myelin retraction.^[^ ^24^ ^]^ As changes in the node length suggestively alters conduction velocity, and node lengthening correlates with the number of days of high performance in the reaching task,^[^ ^25^ ^]^ suggesting its involvement in circuit fine‐tuning during motor learning,^[^ ^23^, ^25^, ^46^ ^]^ we asked whether SPRT learning would also lead to similar changes in the nodes of Ranvier. By immunostaining for Caspr and Nav1.6, we identified the entire structure of a node flanked by two adjacent Caspr‐expressing paranodes (**Figure** **4**a,b), and measured the node length and density. We found that during the reaching task, although the total number of nodes at L6 was unaffected in the iRFA and cRFA, node length was significantly increased in cRFA but not iRFA of learner mice (Figure 4c–f and g–i). We further analyzed the frequency distribution of nodal length in 0.2‐µm bin. We found that there were significantly fewer short nodes (e.g., 0.6 µm in length) and more long nodes (e,g. 1.4 µm in length) in the L6 of cRFA of learner mice, and the distribution curve showed a right‐shifting trend with the median of node length displaying an increased tendency, although the frequency distribution as a whole were not significantly different (Figure 4j,k).

### 2.4 Correlation of Nodal Length Dynamic with Motor Learning Performance

Elongated nodal length has been suggested to facilitate action potential transduction along axons and may enhance related neural circuit and function.^[^ ^23^, ^25^ ^]^ To explore the possible relationship between nodal plasticity and learning performance, we plotted the node length against success rate and the number of high‐performance (over 20% success rate) days, and found a positive linear correlation between them (Figure 4l,m). Since new OL production was suppressed in L6 during SPRT motor learning, the nodal elongation might be mainly induced by pre‐existing myelin sheath, i.e., type 2 OL/myelin plasticity. To further explore the possible relationship between the observed oligodendrogenesis suppression and nodal lengthening during SPRT learning, we plotted the node length against the decreased percentage of CC1^+^EdU^+^ cells. We observed a negative linear correlation of node length with the decrease in CC1^+^EdU^+^ cells (Figure 4n). These results, together with the suppression of oligodendrogenesis, suggest that remodeling of the nodes of Ranvier, i.e., type 2 OL/myelin plasticity, rather than new OL/myelin production, may be preferentially adopted during SPRT learning to achieve better performance.

### 2.5 Blocking Oligodendrogenesis in OPC‐*Myrf*‐cKO Mice Improves Motor Learning Performance in SPRT

To confirm the effects of OL plasticity on motor learning, we crossed *Pdgfrα*‐*CreER^T2^* mice with *Myrf* ^fl/fl^ mice to obtain OPC cell‐specific *Myrf* conditional knockout (OPC‐*Myrf*‐cKO) mice. *Myrf* is an essential transcription factor gene for OPCs involved in their differentiation into mature OLs, and the induction of *Myrf* deletion can efficiently block oligodendrogenesis in adult mice without affecting the pre‐existing OLs and myelin.^[^ ^17^, ^18^ ^]^ To assess the effects of *Myrf* deletion, 8‐week‐old *Pdgfrα‐CreER^T2^*: *Myrf* ^fl/fl^: *Rosa26‐lsl‐tdTomato* (*P‐Myrf* ^fl/fl^ *‐tdT*) mice were treated with tamoxifen for 4 days and perfused 4 weeks later for tissue analysis (Figure S6a, Supporting Information). *P‐Myrf* ^+/+^‐*tdT* and *P‐Myrf* ^fl/+^‐*tdT* littermates were used as control mice. Co‐immunolabeling revealed a dramatic reduction (≈65%) in the number of CC1^+^tdT^+^Sox10^+^ newly formed OLs in *P‐Myrf* ^fl/fl^ *‐tdT* mice compared with controls (Figure S8b,c, Supporting Information), an effect of adult oligodendrogenesis suppression similar to that reported previously.^[^ ^18^, ^19^ ^]^ Moreover, a significant decrease (∼70%) in Myrf^+^tdT^+^Sox10^+^ cell density was observed in *Myrf*‐cKO mice compared to control mice (Figure S8d,e, Supporting Information), suggesting an efficient deletion of *Myrf* upon tamoxifen induction. As there was no difference between *P‐Myrf* ^+/+^‐*tdT* and *P‐Myrf* ^fl/+^‐*tdT* mice (Figure S8c,e, Supporting Information), *Pdgfrα‐CreER^T2^*: *Myrf* ^fl/+^ mice were used as control (*Myrf*‐CTL) groups for further studies.

**Figure 5:**
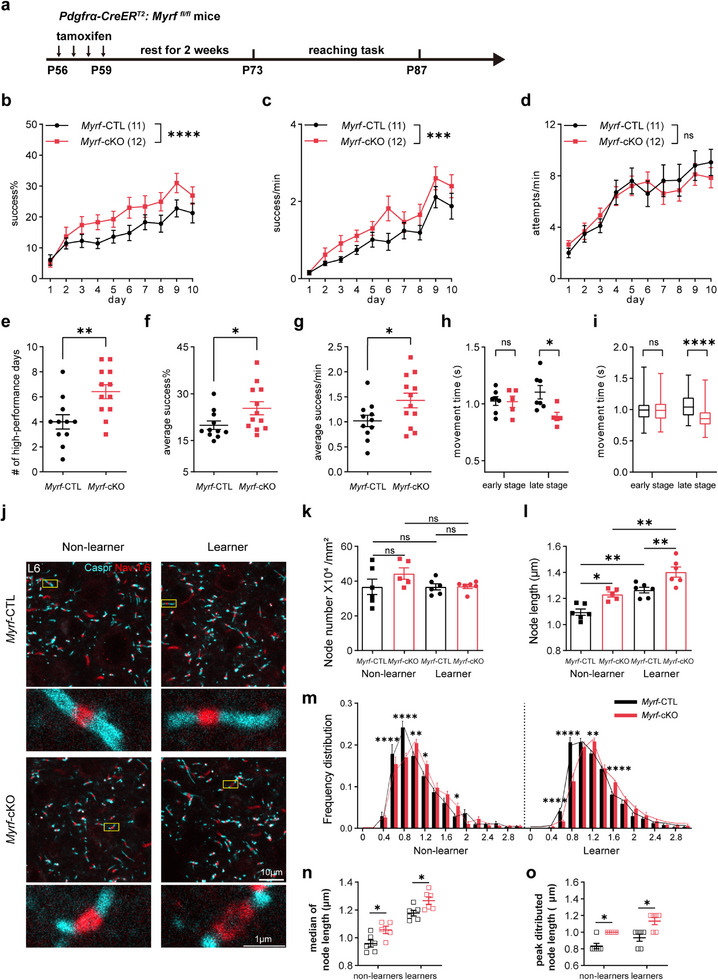
Blocking oligodendrogenesis in OPC‐*Myrf*‐cKO mice improves motor learning performance in SPRT. A) Experimental paradigm for *Myrf* deletion prior to reaching the task. B) Daily success rate in *Myrf*‐cKO (*Pdgfrα‐CreER^T2^: Myrf* ^fl/fl^, n = 11 mice) and *Myrf*‐CTL (*Pdgfrα‐CreER^T2^: Myrf* ^fl/+^, n = 12 mice) mice. Data are presented as mean ± s.e.m. RM two‐way ANOVA: genotype factor, F (1, 210) = 20.24, p < 0.0001; time factor, F (9, 210) = 10.81, p < 0.0001. c Success trials per minute. n = 11 or 12 mice. Data are presented as mean ± s.e.m. RM two‐way ANOVA: genotype factor, F (1, 210) = 15.62, p = 0.0001, time factor, F (9, 210) = 19.58, p < 0.0001. d) Attempts per minute. n = 11 or 12 mice. Data are presented as mean ± s.e.m. RM two‐way ANOVA: genotype factor, F (1, 210) = 0.2020, p = 0.6536, time factor, F (9, 210) = 12.21, p < 0.0001. e) Number of high‐performance days in *Myrf*‐CTL and *Myrf*‐cKO learners. n = 11 or 12 mice. Data are presented as mean ± s.e.m. Mann‐Whitney test, p = 0.0077. f) Average success rate in *Myrf*‐CTL and *Myrf*‐cKO learners. n = 11 or 12 mice. Data are presented as mean ± s.e.m. Unpaired two‐tailed t test, t = 2.122, df = 21, p = 0.0459. g) Average success trials per minute in *Myrf*‐CTL and *Myrf*‐cKO learners. n = 11 or 12 mice. Data are presented as mean ± s.e.m., unpaired two‐tailed t test, t = 2.173, df = 21, p = 0.0414. h Average movement time per mice between genotypes. n = 7 or 5 mice. Data are presented as mean ± s.e.m. Unpaired two‐tailed t‐test, at early stage, t = 0.0991, df = 10, p = 0.9230, at late stage, t = 2.764, df = 10, p = 0.012. Data are presented as mean ± s.e.m. i) Box plot of movement time. 75 and 105 success trials for early and late stage from 7 control mice, and 57 and 70 success trials for early and late stage from 5 cKO mice. Boxes show median with 25%–75% percentile. Mann‐Whitney nonparametric test: on early stage, p = 0.9206, on late stage, p < 0.0001. j) Representative staining image of Caspr and Nav1.6 of *P‐Myrf* ^fl/fl^ mice and control littermates. K) Node density in L6 of RFA, n = 5 or 6 mice. Data are presented as mean ± s.e.m. One‐way ANOVA analysis with Bonferroni's post hoc test, F (3, 19) = 1.470, p = 0.2543. ns, no significance, p > 0.05. l Node length in L6 of RFA, n = 5 or 6 mice. Data are presented as mean ± s.e.m. One‐way ANOVA analysis with Bonferroni's post hoc test. F (3, 19) = 21.13, p < 0.0001. *Myrf*‐CTL (non‐learner) versus *Myrf*‐cKO (non‐learner), adjusted p = 0.0140, *Myrf*‐CTL (non‐learner) versus *Myrf*‐CTL (learner), adjusted p = 0.0014, *Myrf*‐cKO (non‐learner) versus *Myrf*‐cKO (learner), adjusted p = 0.0019, *Myrf*‐CTL (learner) versus *Myrf*‐cKO (learner), adjusted p = 0.0083. m Frequency distribution of node length (from 0 µm to 3 µm) in 0.2‐µm intervals, n = 5 or 6 mice. Data are presented as mean ± s.e.m. Lines are frequency distribution curves. Scheirer‐Ray‐Hare nonparametric test: non‐learner groups, F (genotype factor) = 0.137, p = 0.712, F (genotype × node length, interaction effect) = 5.069, p < 0.0001; learner groups, F (genotype factor) = 0.029, p = 0.865, F (genotype × node length, interaction effect) = 7.767, p < 0.0001. post hoc Bonferroni's test: in non‐learner groups, 0.6‐µm length, adjusted p < 0.0001, 0.8‐µm length, adjusted p < 0.0001, 1.0‐µm length, adjusted p = 0.008, 1.2‐µm length, adjusted p = 0.031, 1.8‐µm length, adjusted p = 0.031; in learner groups, 0.6‐µm length, adjusted p < 0.0001, 0.8‐µm length, adjusted p < 0.0001, 1.2‐µm length, adjusted p = 0.002, at 1.6‐µm length, adjusted p < 0.0001. n) Median of distribution curves (m. n = 5 or 6 mice per group. Data are presented as mean ± s.e.m. Two‐tailed Mann‐Whitney test: non‐learners, p = 0.0303; learners, p = 0.0260. o) Peak distributed node length of distribution curves (m). n = 5 or 6 mice per group. Data are presented as mean ± s.e.m. Two‐tailed Mann‐Whitney test: non‐learners, p = 0.0152; learners, p = 0.0325. ns, no significance, p > 0.05, * p < 0.05, ** p < 0.01, *** p <0.001, **** p < 0.0001.

We then evaluated the learning ability between *Myrf*‐cKO mice (*P‐Myrf* ^fl/fl^) and control mice (*P‐Myrf* ^fl/+^) (**Figure** **5**a). During the first 2 days, *Myrf*‐cKO mice and control mice exhibited similar success rate, but *Myrf‐*cKO mice gradually appeared to outperform control littermates from day3, with the overrvall learning curve of Myrf‐cKO mice significantly exceeded that of control mice as assessed by the success rate and success trials per minute (Figure 5b,c). To rule out the discrepancy in reaching motivation for food reward, we compared the total attempts per minute on each training day between the two groups and observed the same attempt curve, with an increase in early training stages and reaching a plateau (Figure 5d). Also, *Myrf*‐cKO mice show more high‐performance days over 20% success rate, averaged success rate, and averaged successful trials of 10 sessions (Figure 5e–g). As it has been suggested that in dexterous forelimb task, mice not only achieve higher success rates over time, but also form a stereotyped and efficient movement pattern to retrieve pellets or other reward.^[^ ^39^, ^46^, ^47^ ^]^ To determine whether *Myrf*‐cKO mice had quicker movements, we recorded the training video and extracted the success events (Movie S1, Supporting Information). We quantified the movement time and observed *Myrf*‐cKO mice had a shortened reaching duration, leading to less movement time than their control littermates (Figure 5h,i). Additionally, we measured motor balance ability and anxiety‐like behavior using the rotarod test and open field test 4 weeks post‐tamoxifen treatment. Both genotypes showed similar latency to fall, total distance, and proportion of time spent in the center (%) (Figure S9a–c, Supporting Information), suggesting that deletion of *Myrf* does not influence general motor coordination or locomotion activity. Thus, consistent with the motor learning‐induced suppression of oligodendrogenesis, and the negative correlation data between oligodendrogenesis and reaching performance observed above, the artificial blockade of new OL production in adults specifically enhances the motor learning ability in the SPRT, further arguing for an active role of this OL dynamic in SPRT learning.

The enhanced ability of *Myrf*‐cKO mice to learn the SPRT learning again seemed to be contradictory in light of previously observed impaired performance of the same *P‐Myrf* ^+/+^ mouse strain in a “complex wheel” running task.^[^ ^18^, ^19^ ^]^ To further confirm our hypothesis that type 2 OL plasticity might be preferentially adopted to acquire better performance in SPRT learning, we examined whether there were any changes in node plasticity after *Myrf* deletion. Interestingly, an increase in the node length was observed in L6 of both the non‐learner (untrained intact) and learner (motor trained) *Myrf*‐cKO mice compared to the control group, although node density per se remained unchanged (Figure 5j–l). Similarly, the frequency distribution of node length also changed. There were significantly fewer short nodes (e.g., 0.8 µm in length) in both non‐learner and learner *Myrf*‐cKO mice, and significantly more long nodes in both non‐learner (e,g. 1.8 µm in length) and learner *Myrf*‐cKO (e,g. 1.6 µm in length) mice (Figure 5m). The frequency distribution curves of the node length showed a right‐shifting trend toward longer node length, as revealed by the increase in the median of node length and peak distributed node length (Figure 5n,o). These findings suggest that while *Myrf* deletion in OPCs intrinsically alters nodal structure, the learning‐associated enhancement in *Myrf*‐cKO mice may further involve activity‐dependent modifications. Since we have observed a negative linear correlation between the decrease in OL generation in L6 and motor learning performance (Figure 3), we analyzed the correlation further in *Myrf*‐cKO and *Myrf*‐CTL learner mice. With the overall result of the inhibited CC1^+^tdT^+^Sox10^+^ cell density in L6 of cRFA (Figure S8f,g, Supporting Information) in *Myrf*‐cKO mice, we observed a negative linear correlation of the CC1^+^tdT^+^Sox10^+^ cell density with the success rate and the number of high‐performance days in both *Myrf*‐cKO learners and *Myrf*‐CTL learners (Figure S8h,I, Supporting Information). Thus, these results suggest that the inhibition of new OL production and the facilitated node remodeling in *Myrf*‐cKO mice mimics the intrinsic OL dynamics/plasticity of wildtype (WT) mice during the SPRT learning phase and may underlie their superior performance.

### 2.6 Greater Increment of Task‐Related Neuronal Activity in Myrf‐cKO Mice During Motor Learning

**Figure 6:**
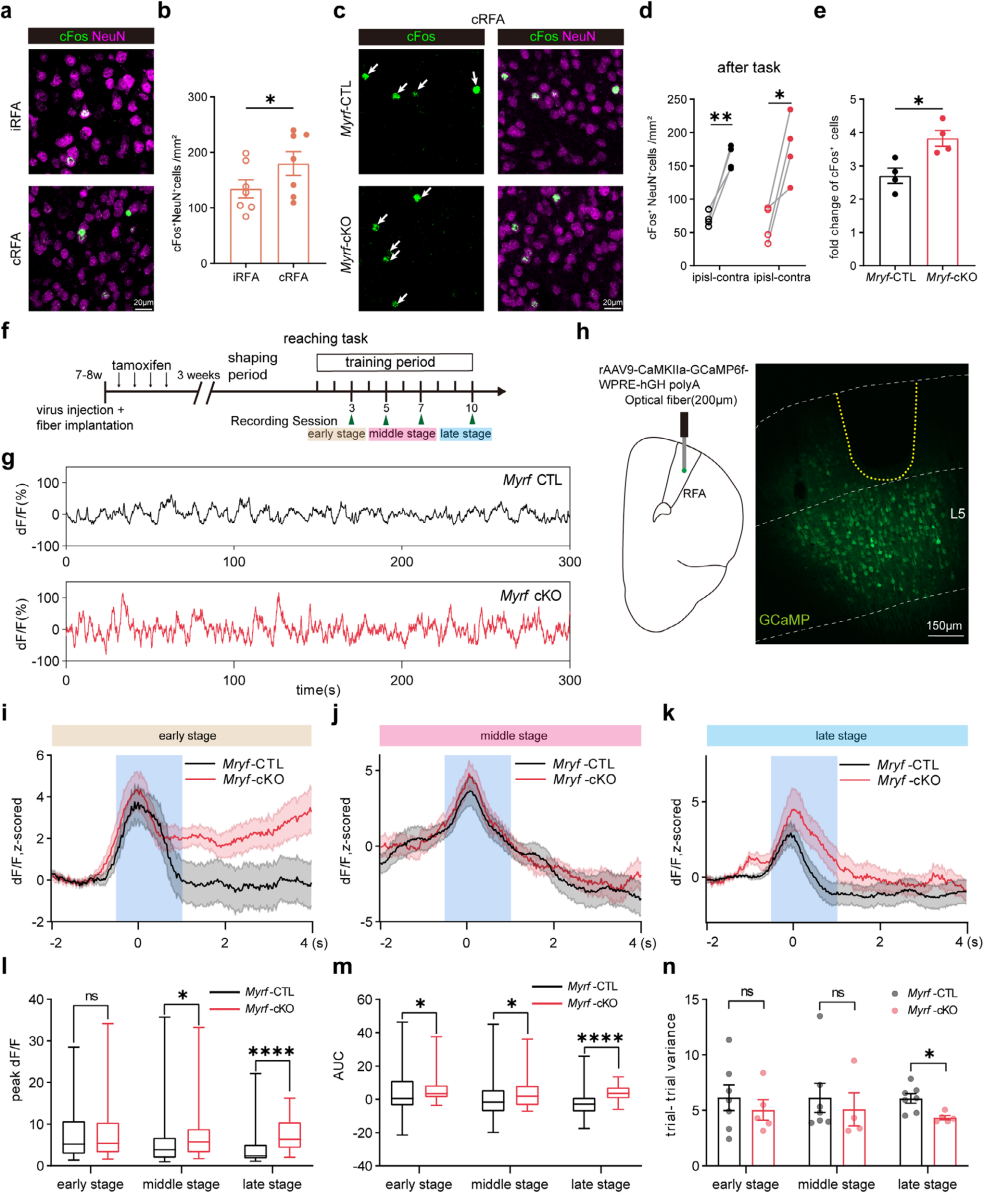
Greater increment of task‐related neuronal activity in *Myrf*‐cKO mice during motor learning. A) Representative image of cFos (green) and NeuN (magenta) in iRFA and cRFA region after reaching task. B) Quantification of cFos^+^NeuN^+^ cell density in learners. Data are presented as mean ± s.e.m. iRFA region: 134.3 ± 16.39, cRFA region: 180.1 ± 21.30. Paired two‐tailed t test: t = 3.149, df = 6, p = 0.0306, n = 7 mice per group. C) Representative image of cFos (green) and NeuN (magenta) in *Myrf*‐cKO and control mice in iRFA and cRFA region after the final training session. D) Quantification of cFos^+^NeuN^+^ cell density in *Myrf*‐cKO and control mice, n = 4 mice per group. Data are presented as mean ± s.e.m. In *Myrf*‐cKO group, iRFA: 62.96 ± 13.46, cRFA: 176.63 ± 24.62, t = 7.899, df = 3, p = 0.0042; in control littermates (*Myrf*‐CTL), iRFA: 69.96 ± 5.45, cRFA: 162.55 ± 8.90, t = 4.027, df = 3, p = 0.0275. Paired two‐tailed t‐test. E) Fold change of cFos^+^NeuN^+^ cell density relative to iRFA in *Myrf*‐cKO and control mice, n = 4 mice per group. Data are presented as mean ± s.e.m. Unpaired two‐tailed t test: t = 3.410, df = 6, p = 0.0143. f) Experimental paradigm for calcium dynamics recording and reaching task. G) Representative calcium signal in a 5‐min behavioral epoch of *Myrf*‐cKO (red) and control (black) mice in learners. ∆F/F is Z‐scored. H) Schematic drawing of virus injection and fiber implantation (left) and image of GCaMP fluorescence expression (right). The yellow dashed line denotes fiber location. I–k) Change of calcium fluorescence (∆F) during movement epochs in a 6‐second time window of *Myrf*‐cKO (red) and control (black) learners. Shading represents s.e.m., 0s represents lift onset, blue area (‐0.5s to 1.5s) shows task‐related area for calculating peak values and AUC. L) Peak ∆F comparison of *Myrf*‐cKO (red) and control (black) learners in 3 training stages. Boxes show median and 25%–75% percentile, whisker length represents maximum and minimum values. In early stage, p = 0.707, in middle stage, p = 0.047, late stage, p < 0.0001. Mann‐Whitney nonparametric test. M) AUC (the area under the curve) comparison of *Myrf*‐cKO (red) and control (black) learners in three training stages. Boxes show median and 25%‐75% percentile, whisker length represents maximum and minimum values. In early stage, p = 0.047, in middle stage, p = 0.025, late stage, p < 0.0001. Mann‐Whitney nonparametric test. N) Trial variance in *Myrf*‐cKO (n = 4 or 5 mice) and control (n = 7 mice) learners’ group. Data are presented as mean ± s.e.m. Early stage, t = 0.7052, df = 10, p = 0.496, middle stage, t = 0.4990, df = 9, p = 0.629, late stage, t = 3.148, df = 10, p = 0.010, unpaired two‐tailed t test. ns, no significance, p > 0.05, * p < 0.05, ** p < 0.01, **** p < 0.0001.

In dexterous forelimb motor tasks, the excitatory neurons exhibit a high degree of dynamism during learning. Task‐related neurons increase and develop into stable movements‐related neurons with practice, and the emergence of task‐specific and movement‐encoding L5 neurons exhibits more reproducible activity and less variable firing in the grasp task, leading to improved motor control and execution.^[^ ^48^ ^]^ Previous studies have suggested that learning‐induced myelin retraction extends the nodes of Ranvier to alter conduction velocity;^[^ ^25^ ^]^ however, how OL/myelin plasticity ultimately modulates motor output signal remains unclear. Therefore, we investigated whether repressed oligodendrogenesis and elongated node length in RFA in *Myrf*‐cKO mice would lead to any changes in neuronal activity in this region during SPRT learning. We first performed cFos labeling of immediately activated neurons within 90 min of the last training session of the SPRT. In learner mice, we observed a dramatic increase in the number of cFos^+^ activated neurons in the cRFA compared with the iRFA (**Figure** **6**a,b), indicating that the forelimb area was activated after reaching the task. A similar increase was also observed in *Myrf*‐cKO mice (Figure 6c,d), and the fold change in cFos^+^ cell numbers in cRFA relative to iRFA was significantly higher in cKO mice (Figure 6e), suggesting that a greater group of task‐specific neurons was recruited in *Myrf*‐cKO mice to acquire better performance.

To better investigate the possible differences in task‐related neuronal activity between *Myrf*‐cKO and control mice, we performed fiber photometry recordings in behaving mice. We injected adeno‐associated virus (AAV) encoding the calcium indicator GCaMP6f driven by an excitatory neuronal promoter (*CaMKIIα*) into the cRFA (L5) of both *Myrf*‐cKO and control mice, followed by fiber implantation at the same site and tamoxifen injection for 4 days to induce *Cre* recombination. After 3 weeks of recovery and viral expression, the mice were subjected to the reaching task. The 10‐day training session was divided into early stage (days 1–3), middle stage (days 5–7), and late stage (days 8–10), and we sampled calcium activity signals every 2 or 3 days while simultaneously recorded behavioral videos to determine the timing of execution. (Figure 6f,g). The injection and plantation locations were verified by post‐hoc histology (Figure 6h). We aligned signals with behavioral videos and plotted z‐score ΔF in successful reach trials (Figure 6i–k). The peak of the z‐score occurred at the timing of lift onset suggesting that neurons were activated in the preparation period, with most successful trials completed in 1.5 s (Figure 5h,i). We set a 2 s epoch ranging from 0.5 s before lift onset to 1.5 s after that as the task window (Figure 6i–k). The area under the curve (AUC) and peak z‐score were calculated at different stages to estimate overall neuronal activity.^[^ ^49^, ^50^ ^]^ Although the peak z‐score and AUC were similar between both genotype mice at the early stage, we observed a significantly higher measurement in *Myrf*‐cKO mice than in their control littermates at the middle and late stages (Figure 6l,m, control: n = 39 trials of early stage,76 trials of middle stage, and 69 trials of late stage from 7 mice, *Myrf*‐cKO: 39 trials of early stage, 40 trials of middle stage, and 51 trials of late stage from 5 mice). We further analyzed the standard deviation of the AUC in each mouse as a trajectory variance and found that the variance of *Myrf‐*cKO mice was reduced compared with control mice in the late stage (Figure 6n), suggesting increased stability across trials and association with more stereotypy in cKO mice. These results, together with the increased number of cFos^+^ cells mentioned above, suggest that during the SPRT, *Myrf*‐cKO mice gradually develop greater task‐related activity with less variation and greater synchronized neuronal population dynamics in the cRFA at the end of the learning phase.

### 2.7 Prolonged Oligodendrogenesis During the Motor Memory Consolidation Phase

**Figure 7:**
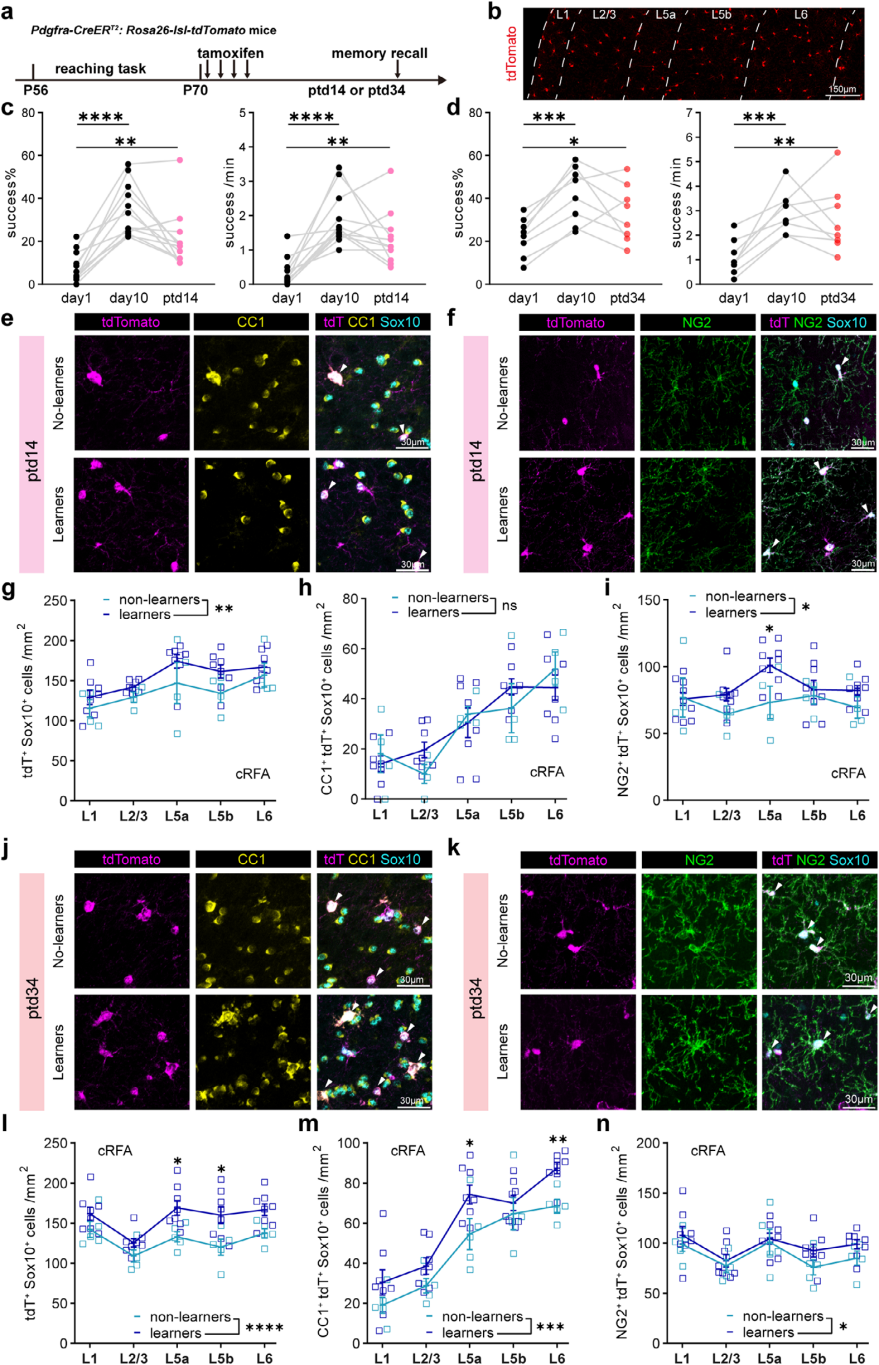
Prolonged oligodendrogenesis in cRFA during motor memory consolidation phase. A) Experimental timeline to trace OPC dynamics during motor memory. B) Cortical layers identification with tdTomato fluorescence. C) Behavioral performance across learning and memory recall (post‐tamoxifen day14, ptd14). Aligned grey lines represent individual mice, n = 11 mice. For success rate (left), day 1 versus day 10, adjusted p < 0.0001, day 1 versus ptd14, adjusted p = 0.0039; for success trials per min, day 1 versus day 10, adjusted p < 0.0001, day 1 versus ptd14, adjusted p = 0.0047. RM one‐way ANOVA with post hoc Dunnett's test. d) Behavioral performance across learning and memory recall (post‐tamoxifen day14, ptd34). n = 8 mice. For success rate (left), day 1 versus day 10, adjusted p = 0.0005, day 1 versus ptd34, adjusted p = 0.0320; for success trials per min, day 1 versus day 10, adjusted p = 0.0004, day 1 versus ptd34, adjusted p = 0.0020. RM one‐way ANOVA with post hoc Dunnett's test. e,f) Representative image of tdT (magenta), CC1 (yellow), and Sox10 (blue) in non‐learners and learners at ptd14 (e) and immunolabeling of tdT (magenta), NG2 (green) and Sox10 (blue) at ptd14 (f). Arrowheads denote tdT^+^CC1^+^Sox10^+^ cells (e) and tdT^+^NG2^+^Sox10^+^ cells (f). g,h) Quantification of tdT^+^Sox10^+^ cells number (g) and CC1^+^tdT^+^Sox10^+^ cells number (h) in cRFA of learners (n = 8 mice per layer) and non‐learners (n = 4 mice per layer) at ptd14. Data are presented as mean ± s.e.m. (g) non‐learners versus learners (training factor): F (1, 50) = 7.490, p = 0.0086; (h) training factor: F (1, 50) = 0.03723, p = 0.8478. Two‐way ANOVA analysis with Šídák's test. i) Quantification of NG2^+^tdT^+^Sox10^+^ cells number in cRFA of learners (n = 9 mice) and non‐learners (n = 4 mice) at ptd14. Data are presented as mean ± s.e.m. Two‐way ANOVA with Šídák's multiple comparisons test: training factor: F (1, 55) = 6.525, p = 0.0134. L5a: adjusted p = 0.0437. j,k) Representative image of tdT (magenta), CC1 (yellow) and Sox10 (blue) in non‐learners and learners at ptd34 (j) and immunolabeling of tdT (magenta), NG2 (green) and Sox10 (blue) at ptd34 (k). l,m) Quantification of tdT^+^Sox10^+^ cells number (l) and CC1^+^tdT^+^Sox10^+^ cells number (m) in learners (n = 8 mice per layer) and non‐learners (n = 5 mice per layer) at ptd34. Data are presented as mean ± s.e.m. Two‐way ANOVA with Šídák's multiple comparisons test: (l) training factor: F (1, 55) = 26.21, p < 0.0001. In L5a, adjusted p = 0.0124, in L5b, adjusted p = 0.0108, in L6, adjusted p = 0.0359. (m) training factor: F (1, 55) = 16.00, p = 0.0002. In L5a, adjusted p = 0.0442, in L6, adjusted p = 0.0067. n) Quantification of NG2^+^tdT^+^Sox10^+^ cells number in learners (n = 8 mice per layer) and non‐learners (n = 5 mice per layer) at ptd34. Data are presented as mean ± s.e.m. Two‐way ANOVA, training factor: F (1, 55) = 4.256, p = 0.0439. ns, no significance, p > 0.05, * p < 0.05, ** p < 0.01, *** p <0.001, **** p < 0.0001.

Once a motor skill has been acquired, it does not require constant practice to maintain, since the formation of new dendritic spines provides a structural basis for memory consolidation and long‐term retention.^[^ ^38^, ^51^ ^]^ In the above experiments, we have explored the changes in oligodendrogensis across cortical layers during SPRT learning. We next asked what would be the situation during the motor memory consolidation phase. We continue to use the transgenic strategy to label OPCs and tracking their differentiation (**Figure** **7**a). To specifically track oligodendrogenesis during the memory consolidation phase, Cre recombination was induced 24 h after the completion of the 13‐ to 15‐day SPRT learning. Mice are allowed to rest for 14 or 34 days post‐tamoxifen injection (ptd14, ptd34) before being reintroduced to a rehearsal session to assess their skill retention/recall. They were subsequently perfused for histological assessment of changes in oligodendrogenesis (Figure 7a). In line with previous reports,^[^ ^38^, ^40^, ^51^ ^]^ the learners showed better success rates and success trials per minute at both ptd14 and ptd34 than when they were first introduced (Figure 7c,d). Our cell counting analysis revealed a significant increase in the number of tdT^+^Sox10^+^ cells at ptd14 in the overall cRFA (Figure 7b,e–g), and an increase in NG2^+^tdT^+^Sox10^+^ cells, particularly in L5a of the cRFA in learner mice (Figure 7i), indicating that the division of OPCs accelerates after learning. We have previously identified *Enpp6* as a novel marker and powerful sensor of newly‐forming/formed OLs, which can maintain a high level expression for ≈1 week after differentiation into CC1^+^ OLs and is subsequently downregulated.^[^ ^19^ ^]^ We then quantified the *Enpp6* ^+^ cells by in situ hybridization and did observed an increase in cRFA in learner mice (Figure S10a–c, Supporting Information), suggesting that there is already enhanced OPC differentiation and oligodendrogenesis during this early period after motor training. These dynamic changes in OPC proliferation and differentiation were not observed in the iRFA (Figure S10d,e, Supporting Information).

With a longer period of post‐training memory consolidation (ptd34), both tdT^+^Sox10^+^ and NG2^+^tdT^+^Sox10^+^ cell densities remained elevated (Figure 7k,l,n), and the number of CC1^+^tdT^+^Sox10^+^ newly produced OL cells increased in the cRFA of learner mice (Figure 7j,m), demonstrating an accelerated accumulation of oligodendrogenesis during this long period of motor memory consolidation. This increase was not observed in the iRFA region (Figure S10f,g, Supporting Information). These results show that, in contrast to the suppressed oligodendrogenesis in the cRFA during the motor skill learning phase, new OL production was enhanced during the following consolidation phase, indicating the involvement of de novo OL generation (type1 OL plasticity) in the regulation of motor memory and related neural circuits.

### 2.8 Correlation of OL Dynamic With Motor Skill Retention

**Figure 8:**
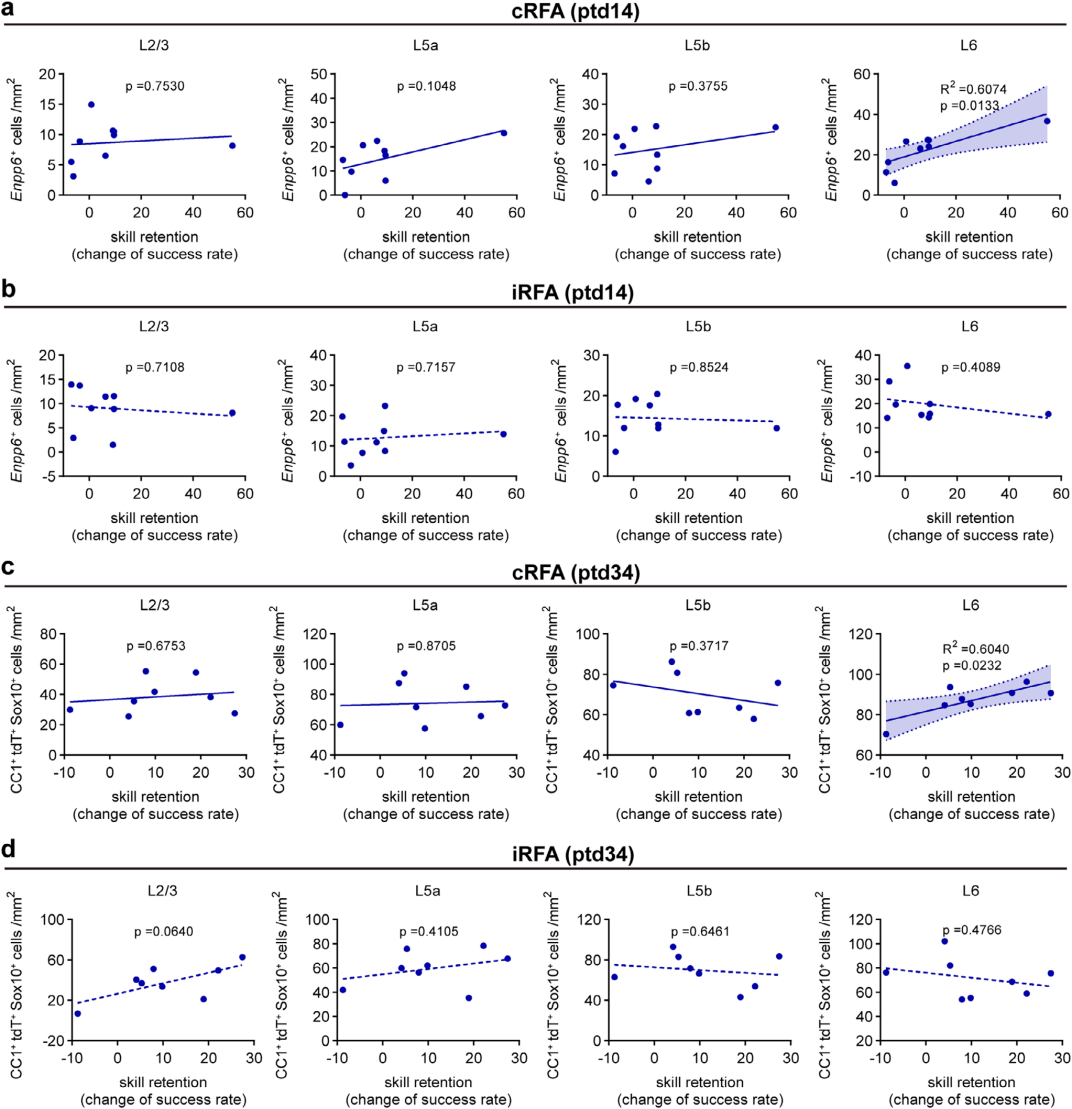
The correlation between OL dynamics and skill retention during the memory consolidation phase. a,b) Correlation between *Enpp6* ^+^ cell density with skill retention (success rate of ptd14 relative to the first training day) for individual mice (n = 9 learners). c,d) Correlation between CC1^+^tdT^+^Olig2^+^ cell density with skill retention (success rate of ptd34 relative to the first training day) for individual mice (n = 8 learners). Pearson correlation, no significance, p > 0.05, * p < 0.05.

To investigate whether the extent of OL production is related to the retention of reaching skills, we plotted the density of *Enpp6* ^+^ cells, which a sensor of newly‐forming/formed OLs at ptd14, against the change in success rate in the rehearsal session versus the first training session of the learner group. We found that across the cortical layers in the cRFA, *Enpp6* ^+^ cells in L6 were positively correlated with the change of success rate, whereas the correlations across other layers (L2/3‐L5b) were not significant (**Figure** **8**a). The correlations were not obvious for iRFA across the different layers, suggesting that changes in the L6 of cRFA were driven by the dominant limb (Figure 8b).

We further plotted the change of success rate at ptd34 against the density of CC1^+^tdT^+^Sox10^+^ cells in contralateral and ipsilateral RFA across different layers. Consistently, the density of CC1^+^tdT^+^Sox10^+^ cells, displayed a linear relationship with the change of success rate in L6 of cRFA (Figure 8c), but not in L2/3‐L5b or iRFA (Figure 8c,d). These data demonstrate a specific linear correlation between oligodendrogenesis in the L6 of the cRFA and the memory consolidation of motor skill, indicating an opposite effect of oligodendrogenesis on skill retention compared to skill acquisition during the training phase.

### 2.9 Blocking Oligodendrogenesis in *Myrf*‐cKO Mice After SPRT Learning Impairs Motor Skill Maintenance

**Figure 9:**
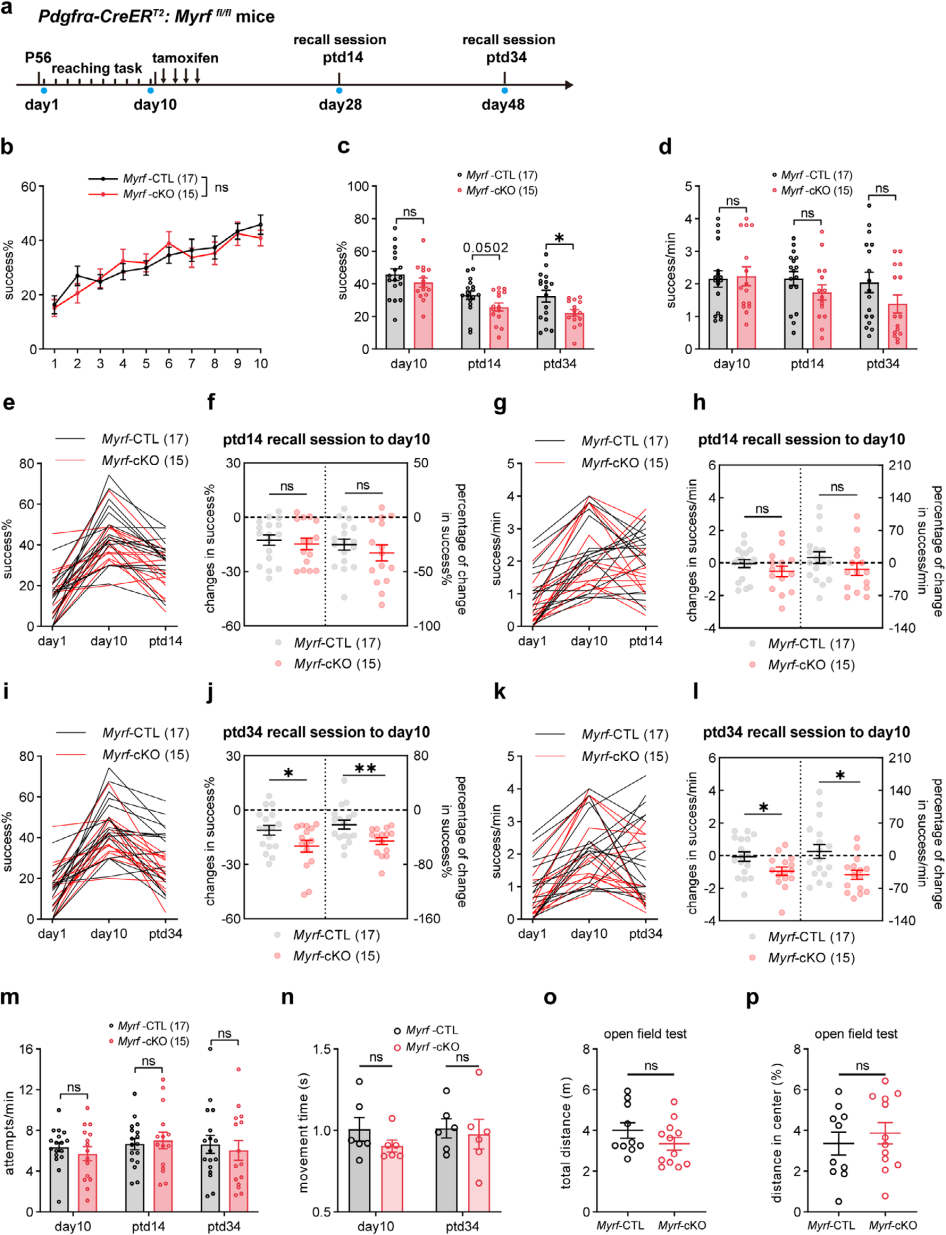
Blocking oligodendrogenesis in *Myrf*‐cKO mice after SPRT learning impairs motor skill maintenance. A) Experimental timeline to assess motor memory after *Myrf* conditional knockout. B) Daily success rate of *P‐Myrf* ^fl/+^ (*Myrf*‐CTL, 17 mice) and *P‐Myrf* ^fl/fl^ (*Myrf*‐cKO, 15 mice) groups during 10‐day training task before *Myrf* deletion. Data are presented as mean ± s.e.m. Repeated‐measured two‐way ANOVA: time factor, F (3.567, 107.0) = 18.23, p < 0.0001, genotype factor, F (1, 30) = 0.04203, p = 0.8389. c) Average success rate across learning and motor memory periods in *P‐Myrf* ^fl/+^ (*Myrf*‐CTL, 17 mice) and *P‐Myrf* ^fl/fl^ (*Myrf*‐cKO, 15 mice) groups. Data are presented as mean ± s.e.m. On day10, *Myrf*‐CTL mice, 45.78% ± 3.60, *Myrf‐*cKO mice, 40.89% ± 2.85, t = 1.045, df = 30, p = 0.3041; on ptd14, *Myrf*‐CTL mice, 33.17% ± 2.54, *Myrf‐*cKO mice, 25.84% ± 2.51, t = 2.040, df = 30, p = 0.0502; on ptd34, *Myrf*‐CTL mice, 32.51% ± 3.61, *Myrf‐*cKO mice, 22.14% ± 2.04, t = 2.412, df = 30, p = 0.0221. Unpaired two‐tailed t‐test. d) Average success trials per min across learning and motor memory periods in *Myrf*‐CTL (17 mice) and *Myrf*‐cKO (15 mice) groups. Data are presented as mean ± s.e.m. On day10, *Myrf*‐CTL mice, 2.16 ± 0.25, *Myrf*‐cKO mice, 2.24 ± 0.29, t = 0.2080, df = 30, p = 0.8366; on ptd14, *Myrf*‐CTL mice, 2.17 ± 0.21, *Myrf‐*cKO mice, 1.74 ± 0.23, t = 1.354, df = 30, p = 0.1858; on ptd34, *Myrf*‐CTL mice, 2.04 ± 0.31, *Myrf*‐cKO mice, 1.38 ± 0.28, t = 1.569, df = 30, p = 0.1271. Unpaired two‐tailed t‐test. e) Individual comparison of changes in success rate in *Myrf*‐CTL (n = 17 mice) and *Myrf*‐cKO (n = 15 mice) groups. F) Changes and percentage of change in success rate in recall session (ptd14) relative to day 10 in *Myrf*‐CTL (n = 17 mice) and *Myrf*‐cKO (n = 15 mice) groups. Data are presented as mean ± s.e.m. Left, *Myrf*‐CTL, −12.61% ± 2.86, *Myrf*‐cKO, −14.65% ± 3.21. Right, *Myrf*‐CTL, −73.64% ± 5.08, *Myrf*‐cKO, −80.52% ± 7.39. Unpaired two‐tailed t test: changes in success rate: t = 0.4759, df = 30, p = 0.6376; percentage of change in success rate: t = 0.8709, df = 30, p = 0.3908. g) Individual comparison of changes in success trials per minute in *Myrf*‐CTL (n = 17 mice) and *Myrf*‐cKO (n = 15 mice) groups. H) Changes and percentage of change in success trials per minute in recall session (ptd14) relative to day 10 in *Myrf*‐CTL (n = 17 mice) and *Myrf*‐cKO (n = 15 mice) groups. Data are presented as mean ± s.e.m. Left, *Myrf*‐CTL, −1.70 ± 0.25, *Myrf*‐cKO, −2.8 ± 0.32. Right, *Myrf*‐CTL, 11.29% ± 12.53, *Myrf*‐cKO, −13.68% ± 13.29. Unpaired two‐tailed t test: changes in success trials per minute: t = 1.189, df = 30, p = 0.2438; percentage of change in success trials per minute: t = 1.367, df = 30, p = 0.1818. i) Individual comparison of changes in success rate in *Myrf*‐CTL (n = 17 mice) and *Myrf*‐cKO (n = 15 mice) groups. J) Changes and percentage of change in success rate in recall session (ptd34) relative to day 10 in *Myrf*‐CTL (n = 17 mice) and *Myrf*‐cKO (n = 15 mice) groups. Data are presented as mean ± s.e.m. Left, *Myrf*‐CTL, −11.09% ± 2.69, *Myrf*‐cKO, −19.88% ± 3.28. Right, *Myrf*‐CTL, −21.39% ± 6.67, *Myrf*‐cKO, −45.62% ± 5.22. Unpaired two‐tailed t test: changes in success rate: t = 2.093, df = 30, p = 0.0449; percentage of change in success rate: t = 2.809, df = 30, p = 0.0087. k) Individual comparison of changes in success trials per minute in *Myrf*‐CTL (n = 17 mice) and *Myrf*‐cKO (n = 15 mice) groups. L) Changes and percentage of change in success trials per minute in recall session (ptd34) relative to day 10 in *Myrf*‐CTL (n = 17 mice) and *Myrf*‐cKO (n = 15 mice) groups. Data are presented as mean ± s.e.m. Left, *Myrf*‐CTL, −0.07 ± 0.29, *Myrf*‐cKO, −0.96 ± 0.26. Right, *Myrf*‐CTL, −9.035% ± 14.93, *Myrf*‐cKO, −41.06% ± 9.79. Unpaired two‐tailed t test: changes in success trials per minute: t = 2.274, df = 30, p = 0.0303; percentage of change in success trials per minute: t = 2.725, df = 30, p = 0.0106. m) Attempts per min across learning and motor memory periods in *P‐Myrf* ^fl/+/+^ (*Myrf*‐CTL, n = 17 mice) and *P‐Myrf* ^fl/fl^ (*Myrf*‐cKO, n = 15 mice) groups. Data are presented as mean ± s.e.m. On day10, t = 0.8057, df = 30, p = 0.4267, on ptd14, t = 0.3575, df = 30, p = 0.7232, on ptd34, t = 0.4515, df = 30, p = 0.6548. Unpaired two‐tailed t‐test. n Average movement time in *Myrf*‐CTL and *Myrf*‐cKO mice, n = 6 mice. Data are presented as mean ± s.e.m. On day10, t = 1.257, df = 10, p = 0.2371, on ptd34, t = 0.3292, df = 10, p = 0.7487. Unpaired two‐tailed t‐test. o) Total exploration distance of *Myrf‐*cKO (n = 12 mice) and control mice (n = 10 mice) during the open field test. Data are presented as mean ± s.e.m. Unpaired two‐tailed t test, t = 1.356, df = 20, p = 0.1903. p) Proportion of exploring distance in the center area of *Myrf‐*cKO (n = 12 mice) and control (n = 10 mice) mice. Data are presented as mean ± s.e.m. Unpaired two‐tailed t test: t = 0.6583, df = 20, p = 0.5179. ns, no significance, p > 0.05, * p < 0.05, ** p < 0.01.

To determine whether blocking OL genesis would also affect the motor memory, we first performed a motor recall/rehearsal experiment 14 days after motor learning in *Myrf*‐cKO and control mice that had undergone Cre induction prior to the learning (Figure S11a, Supporting Information). We detected similar success rates and success trials per minute in both groups (Figure S11a,e, Supporting Information), and observed a trend toward a greater decrease in the success trials per minute in the *Myrf*‐cKO mice, though this was not statistically significant (Figure S11c,f,g, Supporting Information). To unambiguously determine the effect of OL genesis specifically on memory consolidation, we induced oligodendrogenesis blockage after motor learning (start of the memory consolidation period), i.e., tamoxifen was injected into *P*‐*Myrf* ^fl/fl^ mice and *P*‐*Myrf* ^fl/+^ mice immediately after the 10‐day motor learning period (**Figure** **9**a). The learning curves of both genotypes were developed similarly over the 10‐day session, reaching a success rate of ≈40% on day 10 (Figure 9b). Because we observed altered oligodendrogenesis in learners at ptd14 and ptd34, we introduced rehearsal sessions at these same time points. At ptd14 (day28), *Myrf*‐cKO mice showed a trend toward lower success rates than control mice, though the difference was not statistically significant (Figure 9c). The number of success trials per minute and the degree of changes in both success rates and success trails per minute compared to day10 were similar between genotypes (Figure 9d,e–h). However, at ptd34 (day48), *Myrf*‐cKO mice exhibited significantly lower success rates than control mice (Figure 9c), and they also exhibited significantly greater declines in motor performance compared to day10 (Figure 9i–l). Indeed, during the 14‐ or 34‐day memory consolidation period, a smaller proportion of *Myrf*‐cKO mice acquired improved performance, whereas a larger proportion of them showed motor skill decline (Figure S11h–k, Supporting Information). No differences in the total attempts and movement time in the retrieval session were observed (Figure 9m,n), and the locomotion ability and anxiety‐like behavior (Figure 9o,p) remained unchanged between the two genotypes, excluding the influence of food motivation and general motor deficits in *Myrf*‐cKO mice. Thus, our results demonstrate that artificial blocking of new OL generation after SPRT learning can lead to impaired motor skill maintenance.

### 2.10 Blocking Oligodendrogenesis in *Myrf*‐cKO Mice After SPRT Learning Impairs Movement‐Related Calcium Dynamics During Rehearsal

**Figure 10:**
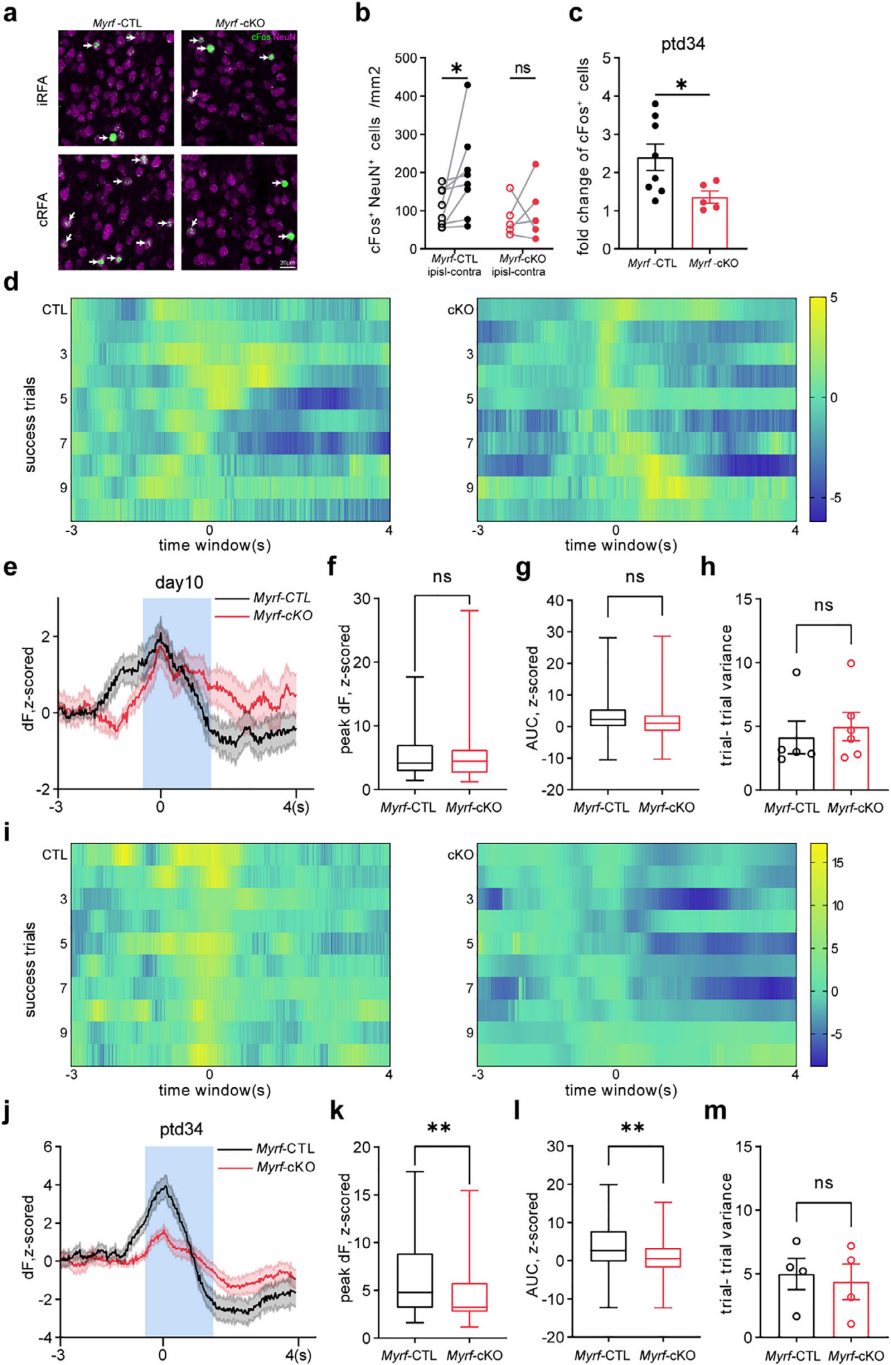
Blocking oligodendrogenesis in *Myrf*‐cKO mice after SPRT learning impairs movement‐related calcium dynamics during rehearsal. A) Representative image of cFos (green) and NeuN (magenta) of *Myrf*‐cKO and control learners in iRFA and cRFA after a rehearsal session. B) Quantification of cFos^+^NeuN^+^ cell density. In *Myrf*‐CTL group, n = 8 mice, iRFA: 107.02 ± 17.34, cRFA: 194.88 ± 41.10, t = 2.151, df = 7, p = 0.0356; in *Myrf*‐cKO, n = 5 mice, iRFA: 79.63 ± 21.55, cRFA: 99.18 ± 34.45, t = 0.4495, df = 4, p = 0.6818, two‐way ANOVA with multiple comparison t‐test. c) Fold change of cFos^+^NeuN^+^ cell density relative to iRFA in *Myrf*‐cKO and *Myrf*‐CTL mice. n = 5 or 8 mice. Data are presented as mean ± s.e.m. Unpaired two‐tailed t test: t = 2.239, df = 11, p = 0.0468. d) Calcium activity on the last training session (day 10) of *Myrf*‐CTL and *Myrf*‐cKO learners before *Myrf* deletion. 10 representative trials are shown for each group in a 7s time window. E) Change of calcium fluorescence (∆F) during movement epochs in *Myrf*‐cKO and control learners. Shading represents s.e.m., 0 s represents lift onset, blue region (−0.5 to 1.5 s) shows task‐related area. F) Comparison of peak ∆F between *Myrf*‐CTL (n = 72 trials from 5 mice) and *Myrf*‐cKO (n = 69 trials from 6 mice) learners. The Boxes show the median and 25%–75% percentile, whisker length represents maximum and minimum values. p = 0.9002, Mann‐Whitney test. g) Comparison of AUC between *Myrf*‐CTL (n = 72 trials from 5 mice) and *Myrf*‐cKO (n = 69 trials from 6 mice) learners. The Boxes show the median and 25%–75% percentile, whisker length represents the maximum and minimum values. p = 0.0642, Mann‐Whitney test. h) Comparison of variance between *Myrf*‐CTL (n = 5 mice) and *Myrf*‐cKO (n = 6 mice) learners. Data are presented as mean ± s.e.m. p = 0.5368. Mann‐Whitney test. i) Calcium activity on retrieval day (ptd34) of *Myrf*‐CTL and *Myrf*‐cKO learners. 10 representative trials are shown in each group. J) Change of calcium fluorescence (∆F) during 7s‐epoch in *Myrf*‐cKO and *Myrf*‐CTL learners. K) Comparison of peak ∆F between *Myrf*‐CTL (n = 54 trials from 4 mice) and *Myrf*‐cKO (n = 59 trials from 5 mice) learners. The boxes show median and 25%–75% percentile, whisker length represents maximum and minimum values. p = 0.0046, Mann‐Whitney test. l) Comparison of AUC between *Myrf*‐CTL (n = 54 trials from 4 mice) and *Myrf*‐cKO (n = 54 trials from 5 mice) learners. The Boxes show the median and 25%–75% percentile, whisker length represents the maximum and minimum values. p = 0.0061, Unpaired two‐tailed t test. m) Comparison of variance between *Myrf*‐CTL (n = 4 mice) and *Myrf*‐cKO (n = 4 mice) learners. Data are presented as mean ± s.e.m. Unpaired two‐tailed t test: t = 0.3305, df = 6, p = 0.7522. ns, no significance, p > 0.05, * p < 0.05, ** p < 0.01.

Motor memory driven by experience or task is allocated to specific layer 5 pyramidal neurons in the M1, termed motor engram neurons, which emerge in the late phase of learning and exhibit enhanced new dendritic spine formation and delayed elimination after a prolonged post‐training period.^[^ ^38^, ^52^, ^53^ ^]^ During the retrieval session, learning‐driven neuronal ensembles were reactivated to direct the reaching attempts. We investigated whether the impaired motor memory in *Myrf‐*cKO mice was caused by failure of neuronal reactivation during rehearsal. To address this problem, we detected cFos expression in L5 after animals were reintroduced to a training session on ptd34 (**Figure** **10**a), the cell density of cFos^+^NeuN^+^ was significantly increased in cRFA in CTL mice. However, *Myrf*‐cKO mice showed impaired cFos activation after a memory rehearsal session (Figure 10b). Fold changes in cFos^+^ cells were also reduced in *Myrf*‐cKO mice (Figure 10c). We also performed fiber photometry recordings to measure the calcium activity of L5 excitatory neurons in temporal sequence. Before Cre recombination, both genotypes showed movement‐related calcium dynamics in the last session (Figure 10d–h), in the rehearsal session, the amplitude of calcium activity was decreased in *Myrf*‐cKO mice, where the OL production was blocked during the memory consolidation period (Figure 10i–l). We also compared trial variation, which is crucial for precise reach attempts; however, *Myrf*‐cKO mice presented a similar trial‐trial variance as *Myrf*‐CTL mice (Figure 10h,m). The deficits in normal task‐related neuronal activation in *Myrf*‐cKO mice, together with the changes in oligodendrogenesis observed above, suggest that new OL production, i.e., type 1 OL plasticity, is required to fine‐tune neuronal activity during the motor memory consolidation phase of the SPRT.

## 3 Discussion

Experience‐dependent adaptive myelin and OL plasticity/dynamic has been shown to be involved in learning and memory in recent years.^[^ ^4^, ^10^, ^11^, ^12^, ^13^, ^14^, ^18^, ^19^, ^21^, ^22^, ^45^ ^]^ These dynamics are brain region and circuit dependent, suggesting that they play a role in modulating neuronal activity and signal communication.^[^ ^54^ ^]^ Exploring different tasks‐induced OL dynamics/plasticity may help us to understand how structural links between associated neurons and brain regions are established to execute certain brain functions. In this study, using both transgenic and chemical tracing strategies, we surveyed the changes in oligodendrogenesis in several different motor‐related brain regions during the learning of a dexterous forelimb motor task of SPRT. We found oligodendrogenesis suppression specifically in L6 of cRFA in learner mice (Figures 1h–k and 2), which was accompanied by nodal lengthening during the motor training phase (Figure 4i–k). Most importantly, we found a linear correlation of behavioral performance with both oligodendrogenesis suppression and (Figure 3) and node remodeling (Figure 4l,m), suggesting that node lengthening, which most likely results from remodeling of the pre‐existing myelin sheath given the suppressed oligodendrogenesis at this stage, and possibly at the cost of this suppression (Figure S8f–i, Supporting Information), was adopted during the SPRT learning phase to achieve better performance. The superior motor performance in SPRT learning (Figure 5b,c,e–g), and the elongated nodes (Figure 5l–o), as well as the elevated neuronal activity (Figure 6e,l,m) observed in *Myrf*‐cKO mice in which oligodendrogenesis was genetically blocked, provide additional evidence supporting this hypothesis.

However, during the motor memory consolidation phase, we observed a prolonged and enhanced accumulation of new OL production in the cRFA of learners, which correlated to the rehearsal performance (Figure 7). Consistently, when Cre activity was induced after the SPRT learning to block oligodendrogenesis during this phase, *Myrf*‐cKO mice showed impaired motor skill rehearsal (Figure 9c), as well as reduced neuronal activity (Figure 10b,c,i–l) compared with control mice, although they had performed just as well during the SPRT learning phase when the *Myrf* gene was intact (Figure 9b), suggesting that new OL genesis is required at this phase. Taken together, our results suggest that the SPRT drives distinct OL dynamics/plasticity in a phase‐dependent manner to fine‐tune task‐related neuronal activity, with a preferential involvement of node lengthening (type 2 OL plasticity) during motor learning and new OL production (type 1 OL plasticity) during memory consolidation. These findings propose a novel model of how type 1 and type 2 OL plasticity are differentially involved in motor learning and consolidation.

Our findings are somewhat unexpected as studies have shown that motor learning, but not consolidation, of the “complex wheel” running task stimulates and requires oligodendrogenesis.^[^ ^18^, ^19^ ^]^ However, this is in good agreement with a study that also showed a similar bidirectional change in oligodendrogenesis during the learning and post‐learning phases using the same SPRT model as ours, but whose observation was mainly focused on the L1‐L3 region of the RFA, which is limited by the depth of observation in two‐photon imaging.^[^ ^24^ ^]^

To specify OL genesis in the motor cortex, we divided the cortex into layers. The results showed heterogeneous properties of OPCs from L1 to L6 in their ability to differentiate (Figure 1j,k,m,n,d,e,j,k,h,m). In particular, adult OPCs in the deep cortex (L5‐L6) showed a much higher degree of intrinsic oligodendrogenesis, suggesting that OPCs in different layers are indeed functionally heterogeneous, although they are evenly distributed in the brain across different cortical layers.^[^ ^55^, ^56^, ^57^, ^58^, ^59^ ^]^ Indeed, there may be several subtypes of OPCs according to their proliferative state, such as proliferative OPCs, quiescent OPCs, and primed OPCs, among which primed OPCs are destined to differentiate or die depending on the circumstances.^[^ ^56^ ^]^ OPC differentiation is controlled by several intrinsic transcription factors such as *Olig2*, *Olig1*, and *Sox10*, as well as many external factors, such as neuronal activity, which may be attributable for experience‐dependent OL plasticity.^[^ ^10^ ^]^ Therefore, the observed regionally distinct oligodendrogenesis may also result from region‐specific neuronal activities and the cues they release, which in turn suggests that neuronal cues may differ not only across brain regions but also between different layers within a given cortical region. Indeed, neuronal classes in the motor cortex are heterogeneous across layers,^[^ ^60^ ^]^ and they may adapt different ways to contact with OPCs and secrete different factors to regulate OPC differentiation,^[^ ^61^, ^62^, ^63^, ^64^ ^]^ although more detailed mechanisms remain to be elucidated and may require more advanced in vivo technologies, such as spatial transcriptomics.

*Myrf*‐cKO mice have been widely introduced to investigate the role of new OL/myelin production (type 1 OL/myelin plasticity) in various learning and memory tasks. However, it is unknown whether *Myrf*‐cKO also affects the type 2 plasticity of pre‐existing OL/myelin, which is also crucial for learning and brain function.^[^ ^23^, ^24^, ^25^ ^]^ It would also be interesting to determine whether there is an intrinsic relationship between new OL/myelin production and pre‐existing OL/myelin remodeling, i.e., between the two main types of OL/myelin plasticity. Interestingly, we here showed that when oligodendrogenesis was artificially blocked in OPC‐*Myrf*‐cKO mice, the length of the node of Ranvier was also altered at least in the RFA region that was examined (Figure 5j–o). Most strikingly, the elongation of node length is linearly correlated with the reduction of oligodendrogenesis and motor skill improvement during SPRT learning (Figure 4l–n), implying a direct link between type 2 and type 1 OL plasticity. From an energy metabolism perspective, OLs and OPCs are among the most energy‐demanding cells in the brain and may lead to competition for energy consumption/distribution between the two types of OL plasticity.^[^ ^63^ ^]^ In fact, the metabolic demands of producing and maintaining myelin make oligodendrocytes highly vulnerable to energetic challenges and environmental changes.^[^ ^65^, ^66^, ^67^ ^]^ In this SPRT model, mice were required to undergo a fasting treatment for 13–15 days during the habituation and formal training/learning period (20 h‐starving before training followed by 4 h‐ad libitum access to food after training per day) to ensure that the mice were motivated to grasp and receive food reward during training, which was not the case in the previous “complex wheel running” model.^[^ ^18^, ^19^ ^]^ We speculate that there was somehow a balance on energy budget spend either on new OL production or nodal elongation (myelin retraction by pre‐existing OLs), and that the transient suppression of oligodendrocyte production might be a trade‐off for node elongation to acquire motor skills during the online phase, i.e., the more energy saving way of type 2 OL plasticity (nodal lengthening and oligodendrogenesis suppression) shall be the optimal strategy and was adopted during the learning phase to acquire and improve motor skill. Training of SPRT task activates neurons in the contralateral RFA, and neuronal firing and neurotransmitter release consume a large amount of energy,^[^ ^68^, ^69^ ^]^ thus inhibition of the new OL production can reduce energy consumption. In addition, as a highly lipid and fatty acid‐rich structure, myelin has been most recently suggested to be an energy reserve in the CNS under certain circumstances (e.g., glucose deprivation).^[^ ^65^ ^]^ Thus, the retraction/degradation of the pre‐existing myelin sheath (type 2 OL plasticity) may not only save energy but also even provide neurons with extra energy via the fatty acid metabolic pathway to support and facilitate their function during the learning,^[^ ^65^ ^]^ when there is an overall energy shortage because of fasting. Moreover, the observed nodal lengthening, which is most likely a result from the retraction/shortening of the pre‐existing myelin sheath (as the new OL/myelin production was suppressed during this period), can simultaneously accelerate axonal impulse conduction, facilitate the rapid transmission of information between brain regions and reduce neuronal asynchrony.^[^ ^23^, ^25^ ^]^ All the above aspects may contribute to skill improving in the learning phase. Therefore, in consistent with the above speculation, when OL generation was artificially suppressed in *Myrf*‐cKO mice, where the energy budget for new OL production is likely to be saved and can be readily transferred for a preferential energy supply to neurons during learning, it ultimately resulted in their better performance than WT mice (Figure 5b,c,e–g, 56f–i).

During forelimb motor learning, performance and variability evolve with extensive practice, leading to precise and efficient movements. The reinforcement learning rule suggests that variable movements facilitate exploration and allow the realization of diverse movement outcomes in the initial phase, even though they produce many errors and a high signal‐to‐noise ratio, which emerges as an expanding dynamics of neuronal activities.^[^ ^36^, ^48^, ^70^, ^72^ ^]^ This is followed by refinement into similar and fast movements and the emergence of a small population of task‐related neurons with reproducible activity,^[^ ^39^, ^46^, ^47^, ^48^ ^]^ possibly through synchronized grouped neuronal firing. It is well established that synaptic/spine plasticity, either by enhancing the formation of new synapse/spine or by delaying their elimination, is essential for learning and memory, and appears to function consistently during training and consolidation phases.^[^ ^40^, ^41^, ^51^, ^73^, ^74^ ^]^ However, our results suggest that OL/myelin plasticity appears to function differently in these two phases. During the learning phase, type 2 OL plasticity of nodal lengthening was preferentially adopted, as described above, since elongated node length can affect the timing of action potential arrival and may facilitate the firing synchronization of associated neurons within local or cross‐brain regions, ultimately contributing to the increase in task‐related neuronal activity during SPRT learning to improve movement performance.^[^ ^23^, ^25^ ^]^ On the contrary, during the memory consolidation phase, type 1 OL plasticity of new OL/myelin production was preferentially adopted, which is thought to be critical for strengthening and maintaining the experience‐related neural circuit activity,^[^ ^21^, ^22^, ^23^, ^25^ ^]^ as evidenced by the observed enhancement of oligodendrogenesis and its correlation with the rehearsal performance during this period. The finding that artificially blocking of oligodendrogenesis in *Myrf*‐cKO mice during this phase resulted in impaired rehearsal performance and concomitantly reduced task‐related neuronal activity further supports a critical role for type 1 OL plasticity in motor memory consolidation. Again, from an energy metabolism point of view, the energy expenditure required to enhance new OL/myelin production during the consolidation phase can be well supported as the mice are no longer subjected to prolonged fasting as in the motor learning phase. However, how exactly OL plasticity coordinates with neuronal/synaptic plasticity to fine‐tune brain function during learning and memory consolidation remains to be elucidated. As suggested by Hughes and colleagues,^[^ ^24^ ^]^ the increased myelin retraction rate and node length, as well as the reduced de novo new myelination, may leave large gaps in the activated axons during the learning phase, allowing new myelin sheaths to enwrap around them later during the consolidation phase. After reward learning ceases, the need to reinforce the learning‐activated neural circuits and consolidate skills may continue to stimulate OPC division and differentiation to produce new OLs and add more new myelin sheath. Further studies, such as live two‐photon live imaging of the OL/myelin and node dynamics in P‐*Myrf ^fl/fl^‐tdT* and control mice, would provide more interesting and useful information in the future. For example, it could reveal whether the persisting “old oligodendrocytes” in *Myrf*‐cKO mice will continue to lengthen their nodes and/or produce more myelin sheaths per cell in compensation after “sensing” that no new OLs are being produced.

## 4 Conclusion

Recent years have witnessed the emergence of a new form of neural/brain plasticity, i.e., OL/myelin plasticity, with its necessity in many different forms of learning and memory being increasingly reported.^[^ ^9^, ^10^, ^11^, ^12^, ^13^ ^]^ However, compared to the long and intensive studies on the role and mechanism of the traditional synaptic plasticity played in learning and memory, the mechanism by which experience‐dependent OL plasticity/dynamics, and especially the possible different roles between type 1 and type 2 OL/myelin plasticity that may actively play in the different phases of learning and memory, remains to be unknown, though phasic oligodendrogenesis has been recently revealed by two‐photon imaging observation in the superior layers of motor cortex learning the correlation.^[^ ^24^, ^25^ ^]^ Here, we performed a layer‐specific analysis of oligodendrogenesis both in the contralateral and ipsilateral primary motor cortex, as well as in other motor‐related brain regions, in the SPRT model. To triple confirm, we introduced three orthogonal approaches, including two genetic reporter mouse lines and one chemical labelling method. We also conducted causative, analyses and assessed the neuronal activity via calcium recording and cFos quantification. Our results demonstrate bidirectional changes in OL dynamics: suppressed oligodendrogenesis and increased node length were observed during the motor learning phase, while enhanced oligodendrogenesis was observed during the memory consolidation phase. Notably, these changes correlate with the motor learning and rehearsal performance, respectively. We conclude that SPRT drives distinct OL plasticity in a phase‐dependent manner to fine‐tune task‐related neuronal activity, with a preferential involvement of oligodendrogenesis suppression and node lengthening (type 2 OL plasticity) during learning and oligodendrogenesis enhancement (type 1 OL plasticity) during consolidation in SPRT. Our findings reveal striking regional heterogeneity in the regulatory mechanisms governing oligodendrogenesis across different brain regions and specific behavioral (motor) paradigms, providing a novel framework for understanding experience‐dependent OL/myelin plasticity as an active modulator of the neural network and a direct contributor to advanced brain functions such as learning and memory.

## 5 Experimental Section

### Mice

All mice were carried out in accordance with protocols approved by the Animal Care and Use Committee of South China Normal University (SCNU‐BRR‐2021‐022). Mice were kept on a 12‐h light‐12‐h dark schedule with ad libitum access to food and water, aside from training‐related food restriction. All mice were caged in groups (2–5 mice per cage) and age‐matched (no more than 5 days) between experimental groups. *Myrf* ^fl/fl^ mice were from Ben Emery,^[^ ^17^ ^]^ *Pdgfrα‐CreER^T2^* mice were provided by Bill Richardson's laboratory.^[^ ^18^ ^]^ Reporter mice *Rosa26‐lsl‐tdTomato* mice (0 07909) and *NG2‐CreER^T2^* mice (0 08538) were from The Jackson Laboratory. *Pdgfrα‐CreER^T2^*: *Myrf* ^fl/+^: *Rosa26*‐lsl‐*tdTomato* mice were crossed with *Pdgfrα‐CreER^T2^*: *Myrf* ^fl/+^: *Rosa26‐lsl‐tdTomato* mice for *Myrf* knockout test, *Pdgfrα‐CreER^T2^*: *Myrf* ^fl/fl^ *mice* were crossed to *Pdgfrα‐CreER^T2^*: *Myrf* ^fl/+^ for behavior and fiber photometry experiments. Genotypes of all mice were determined by PCR analysis of tail genomic DNA using primers. Male and female mice were used without bias in all experiments.

### Administration of Tamoxifen

To activate Cre recombination, 10mg mL^−1^ tamoxifen (Sigma‐Aldrich, T5648) was dissolved in a mixture of 90% (v/v) coin oil (MedChemExpress, 8001‐30‐7) and 10% (v/v) ethanol absolute (Sangon Biotech, A50737) by sonication at room temperature for 2 h. Mice were injected intraperitoneally at a dose of 100mg kg^−1^ body weight for four consecutive days. For recombination in learning training, tamoxifen was administrated two or three weeks in advance, for rehearsal assays, tamoxifen treatment began one day after training.

### Single Pellet Reaching Task

The single‐pellet reaching task (SPRT) for testing motor learning was conducted as previously described.^[^ ^40^, ^51^ ^]^ Briefly, mice were food‐restricted to 90% of their previous body weight before the start and monitored during the test. The chamber was constructed of Plexiglas with a 1 mm wide slit in the front of the chamber through which the mouse could reach for pellets on a food container. During the shaping session, mice were placed in chambers to familiarize with the environments and reaching outcomes. A shaping session was completed by 20 attempts per mouse or 20 mins, the dominant forelimb was then determined by over 70% reaches using either limb. When limb preference was determined at least twice, mouse finished shaping session. All mice completed within 3–5 sessions. Training sessions lasted 10 days with 30 trials using the dominant forelimb, or 20 mins each day, whichever came first. The training chamber was the same as the shaping chamber, instead of a food holder in the front offering a single pellet per trial, the food holder was 1 cm tall, 1 cm anterior, and 1 mm lateral to the opposite side of the dominant limb. “Success attempt” was defined if the mouse lifted and reached dominant limb, grasped a pellet on the holder and bring it into its mouth (Movie S1, Supporting Information), “Failure attempt” was classified as “reach failure” (mouse reached its paw but did not touch the pellet, Movie S2, Supporting Information), “grasp failure” (mouse touched the pellet but did not grasp it successfully, Movie S3, Supporting Information) and “retrieval failure” (mouse grasped the pellet but losing it when retrieving). Success rate was calculated as the percentage of success attempts divided by total attempts (success attempts plus failure attempts). Restriction diet was conducted throughout the habituation and training period (lasting 13–15 days) to keep mice motivated (20‐h‐starving before training followed by 4‐h‐ad libitum access to food after training). Mice that achieved at least 20% success rate were included in “learners” group, “non‐learners” were kept on the same diet and training condition but lost reach motivation during learning. In the rehearsal session for motor skill recall at post‐tamoxifen day 14 or day 34 (ptd14 or ptd34), learner mice were kept free access to food after the last training session, but were starved for 20h before reintroduced to a rehearsal session consisting of 30 trials or 20 mins, the same as a training session. All reach tasks were performed by an experimenter blind to genotype.

### Open Field Test

Mice were acclimated to the behavior room for more than 1h and then placed into a rectangular open field box (40 × 40 × 30 cm), where they were allowed to explore freely for 15 mins, the light intensity in the box was adjusted to 50 lux to prevent light‐induced anxiety. Exploration behavior was recorded through a camera (30 frames per second). Analysis was performed using Shanghai XinRuan software. The total travelled distance was quantified to assess locomotion ability, and percentage of distance and time in center was analyzed to assess anxiety‐like behavior.

### Rotarod Test

The Rotarod test was performed to test motor coordination condition after long‐term *Myrf* knockout. Mice were placed in 3 cm‐diameter rods (Ugo Basile, 47 650), and the rod was speeded up constantly from 4 rpm to 40 rpm within 5 min, the cutoff time is 300 s. Per mouse was performed 4 trials and was allowed to rest for 10 min between trials. The average duration that mice can hold on the rod was calculated as the latency to fall (s).

All behavioral assays were carried out at daytime and at least 1h before light was on or off (from 9 a.m. to 7 p.m.), experimental apparatuses were wiped with 10% ethanol to avoid smell cues. The experimenter was blind for genotypes and got familiar with mice 2–3 days in advance to reduce stress.

### Perfusion and Section Preparation

Mice were deeply anesthetized with pentobarbital sodium at 100 mg kg^−1^ body weight and perfused transcardially with 30 ml of 0.01 M phosphate‐buffered saline (PBS) followed by 30 ml 4% (w/v) paraformaldehyde (PFA, Sigma‐Aldrich, P6148) in 0.1M PB. For cFos analysis, mice were perfused within 90 min following the reaching task. Brain tissue was removed and post‐fixed in 4% PFA at 4 °C for 48h, followed by cryoprotection in 20% (w/v) and 30% (w/v) sucrose (30mL) (Sangon Biotech, A610498) in 0.1 M PBS at 4 °C in sequence. Brains were frozen in O.C.T. compound (Sakura, 4583) for storage at −80 °C. 20 µm coronal brain slices were sectioned (Leica, CM1950) and collected as floating sections in 24‐well plates (10 slices per well) containing 0.01M PBS and stored at 4 °C for 2–3 days. Slice locations (RFA region: +2.1 mm to +1.7 mm anterior to bregma, CFA: +1.1 mm to ‐0.1 mm AP (anterior‐posterior) to bregma, MT: −0.95 mm to −1.55 mm AP to bregma) were carefully distinguished during collection and confirmed before staining. Fresh sections were conducted for ISH first. For later storage at −20 °C, sections were transferred into 1.5 mL tubes with a mixture of PBS/glycerol (Sigma‐Aldrich, G5516) (1:1, v/v). Since tissue preparation for in situ hybridization (ISH) was trickier but compatible with immunolabeling, PB, PBS, and sucrose solutions were treated with diethyl pyrocarbonate (DEPC, 1:1000, v/v, Sangon Biotech, B600154) and then sterilized.

### In Situ Hybridization

Detailed protocols are available at http://www.ucl.ac.uk/~ucbzwdr/ richardson.htm. Briefly, digoxigenin (DIG)‐labeled riboprobe was transcribed in vitro with T7 promoter DNA templates of *Enpp6*. Sections were incubated in hybridization buffer with *Enpp6*‐probe (1:1000) at 65 °C overnight. Solutions and materials were prepared in RNAse‐free conditions as possible. After washed by washing buffer, sections were blocked in blocking solution and incubated with anti‐DIG antibody conjugated with alkaline phosphatase (AP) (1:800–1:1000 in blocking solution, Roche, 11 903 274 910) overnight at 4 °C. On day 3, hybridization products were visualized by developing with a mixture of nitroblue tetrazolium (NBT, Roche, 11 383 213 001) and 5‐bromo‐4‐chloro‐3‐indolyl phosphate (BCIP, Roche, 11 383 221 001).

### Immunofluorescence

To immunolabeling OPCs, sections were blocked in 0.01 M PBS with 0.3% (v/v) Triton X‐100 and 10% (v/v) goat serum before washed in 0.01 M PBS with 0.3% Triton X‐100, sections were then incubated in blocking solution containing rabbit anti‐NG2 antibodies (1:200 dilution, Sigma‐Aldrich, AB5320), rat anti‐tdTomato (1:500 dilution, asis Biofarm, OB‐PRT017), guinea pig anti‐Sox10 antibodies (1:500 dilution, asis Biofarm, OB‐PGP001) or anti‐Olig2 antibodies (1:500 dilution, asis Biofarm, OB‐PGP040) for 40 h at 4 °C. Following washed 1 h in PBS, sections were incubated in blocking solution with secondary antibodies, goat anti‐rabbit Alexa Flour 488 (1:500, Abcam, ab150077), goat anti‐rat Alexa Flour 555 (1:500, Abcam, ab150158), and goat anti‐guinea pig Alexa Flour 647 (1:500, Abcam, ab150187) for 2 h at room temperature. To immunolabeling oligodendrocytes, sections were treated in citrate antigen retrieval solution (pH 6.0) for 40 mins at 85 °C followed by blocking and antibodies incubation process as above. Primary antibodies contained rat anti‐CC1 (1:200, asis Biofarm, OB‐PRT039) antibody, rabbit anti‐tdTomato antibody (1:500, asis Biofarm, OB‐PRB013) and guinea pig anti‐Sox10 antibody, secondary antibodies contained goat anti‐rat Alexa Flour 488 (1:500, Abcam ab150157), goat anti‐rabbit Alexa Flour 555 (1:500, Abcam, ab150078) and goat anti‐guinea pig Alexa Flour 647; for co‐staining of Myrf, tdT, Sox10, sections were treated with antigen retrieval solution and incubated with primary rabbit anti‐Myrf (1:500, asis Biofarm, OB‐PRB007), rat anti‐tdTomato and guinea pig anti‐Sox10 antibodies. For immunolabeling of Caspr and Nav1.6, sections were incubated in antigen retrieval solution and then incubated with rabbit anti‐Nav1.6 antibodies (1:500, Alamone labs, ASC‐009) and mouse anti‐Caspr antibody (1:200, NeuroMab, 75–001) and corresponding secondary antibodies.

### EdU labeling in vivo

Mice were given 5‐ethynyl‐2′‐deoxyuridine (EdU, Santa Cruz, sc‐284628A) dissolved in drinking water (0.2 mg mL) for 10 days (P46‐P56) before reaching the task. EdU detection was performed after immunolabeling by Alexa Fluor 647 Click‐iT detection kit (Invitrogen, C10340).

### Analysis of reaching movements

A high‐speed (120 frames per second) camera was placed vertically in front to the animal, and the field was adjusted to capture a fixed region containing training chamber. The video was synchronized with the calcium signal. Potplayer software was used to manually label timestamps of videos. A success trial started by lifting onset from floor and ended by pellet at mouth, duration was quantified as movement time. We labeled 180 trials from *Myrf*‐CTL mice (n = 75 trials of early stage and 105 trials of late stage from the same 7 mice) and 137 trials from *Myrf*‐cKO mice (57 trials of early stage and 70 trials of late stage from the same 5 mice) during training session, 159 trials from *Myrf*‐CTL mice (92 trials for day 10 and 67 trials for ptd34 from the same 6 mice) and 153 trials from *Myrf*‐cKO mice (81 trials for day 10 and 72 trials for ptd34 from the same 6 mice) during memory phase. Starting markers were also imported into the calcium analysis software as “event time” to determine the event‐related time window.

### Fiber Photometry

For in vivo imaging experiments, mice aged 7–8 weeks were anesthetized with pentobarbital sodium (100 mg kg^−1^ body weight) and fixed in a stereotaxic instrument (RWD, 68 807) with eyes applied with ointment. After removing brain skin, we located right rostral forelimb area (1.9 mm anterior, 1.2 mm lateral to bregma) and remove a small piece of brain skull using a skull drill (RWD, 78 001), 60 nl of rAAV‐CaMKIIα‐GCaMP6f‐WPRE‐hGH polyA virus (Brain VTA, PT‐0119, 10^12^ vector genomes/mL) was infused at a rate of 50 nl min^−1^ (RWD, R‐480) into L5 (0.8 mm deep to the brain pia) with a glass micropipette. The tip was left for 10 mins at the end of the injection for virus diffusion and to reduce backflow. After that, fiber implants (Nanjing Thinkertech, 200 µm in outer diameter and 2 mm in length) was placed into the same location with 0.7 mm in depth and fixed with dental cement. After surgery, mice were subcutaneously injected dexamethasone sodium to prevent inflammation and recovered at 37 °C heating pad to maintain body temperature. For Cre recombination during motor training, tamoxifen was given at least 1 day after recovery. After 3 weeks for expression, signals were sampled in behaving mice using the fiber photometry system (Nanjing Thinkertech) consisting of 405 nm, 470 nm, and 580 nm light‐emitting diode (LED). The Mouse was gently inserted fiber attached to the system (habituated once before experimental recording) through an optical implant and placed in the behavior apparatus. 470 and 410 nm LEDs were used for GCaMP6f excitation and control signals, respectively. Both emitted signals were captured at 40 Hz. Raw data was collected which contained a 30 s baseline, a 5–10 mins behavior signal and a 30 s offline signal. Data from the left‐dominant forelimb mice was analyzed further in MATLAB. To verify viral expression and implant placement, all animals were perfused for post hoc analysis.

### Fiber Data Processing

Calcium activity data was analyzed using the TripleColorMultiFiberPhotometry Software (Nanjing Thinkertech) in MATLAB 2017b. To reduce the effect of photobleaching caused by long‐term recording session, 410 and 470 nm signals were pre‐processed in “Baseline correction” Module, the correct time was set between 5–10 mins the behavior signal (marked during data acquisition), and lamda index was set on 8. To quantify event‐related calcium activities, we used “Average” module, z‐scored ∆F was calculated as the relative change of the GCaMP6f signal (F(t) during behavior phase to the mean value of the GCaMP signal during baseline (F_0_), compared to the standard deviation of F_0_: $Z-score=\frac{F\left(t\right)-F_{0}}{\sigma_{F_{0}}}$. Baseline was defined as a 2 s period from −1 to −3 s prior to event time. Reach‐related calcium activities were defined between 0.5 s before and 1.5 s after the lift onset. The area under the curve (AUC) and the peak value was calculated to assess movement‐related calcium activities. The AUC was calculated by multiplying the average z‐scored ∆F with the time interval within the reach‐related time range. The deviation of AUC across trials of each mouse was calculated to analyze event variance.

### Histological Quantification

Images for cell counting were acquired with Leica microscopy (DMi8, 20× objective). ImageJ software was used for defining regions of interest and cell quantification. Depth of L1‐L6 was divided, referring to Paxinos and Franklin's mouse brain map (3rd edition), tdTomato^+^Sox10^+^ or tdTomato^+^Olig2^+^ colocalization was manually counted and then merged with CC1 or NG2 for triple co‐labeling. To identify differentiated OL by CC1 expression, OPCs were excluded with weak expression of CC1 and multi‐process morphology.^[^ ^75^ ^]^ For *Enpp6* measurement, only highly expressed *Enpp6* cells, which represents newly differentiating oligodendrocytes, were counted. To measure the length and density of the node of Ranvier, high‐magnification (63x objective, Zeiss, LSM 900) confocal images were acquired at 0.38 µm intervals, and 6 interleaved slices were stacked with maximum intensity in ImageJ subsequent analysis of node length was calculated using a MATLAB script provided by David Attwell.^[^ ^76^ ^]^ At least 3 sections were sampled in each mouse. All analyses were conducted in a blind.

### Statistical Analysis

All statistical analysis were performed using the GraphPad Prism software (v.9) and SPSS software. Normality test was conducted with the Shapiro‐Wilk or Kolmogorov‐Smirnov test (n > 50). Data which passed normality test was represented as mean ± s.e.m., Repeated one‐way ANOVA analysis was used for learning performance of learners, followed by post hoc Tukey's or Dunnett's multiple comparisons test, one‐way ANOVA analysis was used for OL dynamic of *Myrf*‐cKO followed by post hoc Tukey's test, or nodal changes of *Myrf*‐cKO followed by post hoc Bonferroni's test. Two‐way ANOVA analysis was used to compare cell density of L1‐L6 in cortex across groups with Šídák's post hoc test, and daily learning performance in the control and *Myrf*‐cKO group. Scheirer‐Ray‐Hare nonparametric test was applied to frequency distribution curves of the node length, followed by post hoc Bonferroni's test. The Mann‐Whitney nonparametric test was applied to node length metrics of frequency distribution curves. Unpaired Student's t‐test was used to compare cell density and other measurements between groups in a specific area, and nodal density between groups. cFos data were assessed with an unpaired Student's t‐test between groups or a paired Student's t‐test between contra‐ and ipsil‐ cortex. For calcium activity analysis, trial variance which passed the normality test was analyzed with an unpaired Student's t‐test. Peak value and AUC data were not satisfied with normality and represented as median with 25%–75% percentile, these values were compared with the Mann‐Whitney nonparametric test. Significance was reported as ns, no significance, * p and adjusted p < 0.05, ** p and adjusted p < 0.01, *** p and adjusted p < 0.001, **** p and adjusted p < 0.0001.

## Conflict of Interest

The authors declare no conflict of interest.

## Author Contributions

L.X. acquired funding, supervised the research, and provided research resources. L.X., S.W., H.L., and G.L. conceptualize and develop methodology. S.W. carried out most of the experiments and collected the data with help from N.X., W.W., and also from Y.H., Y.Y., L.Z., Y.Z., and Y.H. S.W. and L.X. wrote the manuscript, all other authors contributed to the review and editing. N.X. and W.W. contributed equally.

## Supporting information



Supporting Information

Supplemental Movie 1

Supplemental Movie 2

Supplemental Movie 3

## Data Availability

The data that support the findings of this study are available from the corresponding author upon reasonable request.
