# Supplementary material for: Forelimb Motor Learning and Memory Consolidation Drives Distinct Oligodendrocyte Plasticity to Regulate Task‐related Neuronal Activity
Source: Adv Sci (Weinh). 2025 Sep 30;12(48):e05367. doi: 10.1002/advs.202505367 (PMC12752644; doi:10.1002/advs.202505367)
Supplement: Supplementary file 1 — Supporting Information [file ADVS-12-e05367-s001.docx]

Supplemental Figures

**Forelimb motor learning and memory consolidation drives distinct oligodendrocyte plasticity to fine-tune task-related neuronal activity**

*Shuming Wang, Nuo Xu, Wenwen Wang, Yongxiang He, Yuqian Yang, Liuning Zhang, Yanping Zou, Yuehua He, Huiliang Li, Liang Gao and Lin Xiao*

**
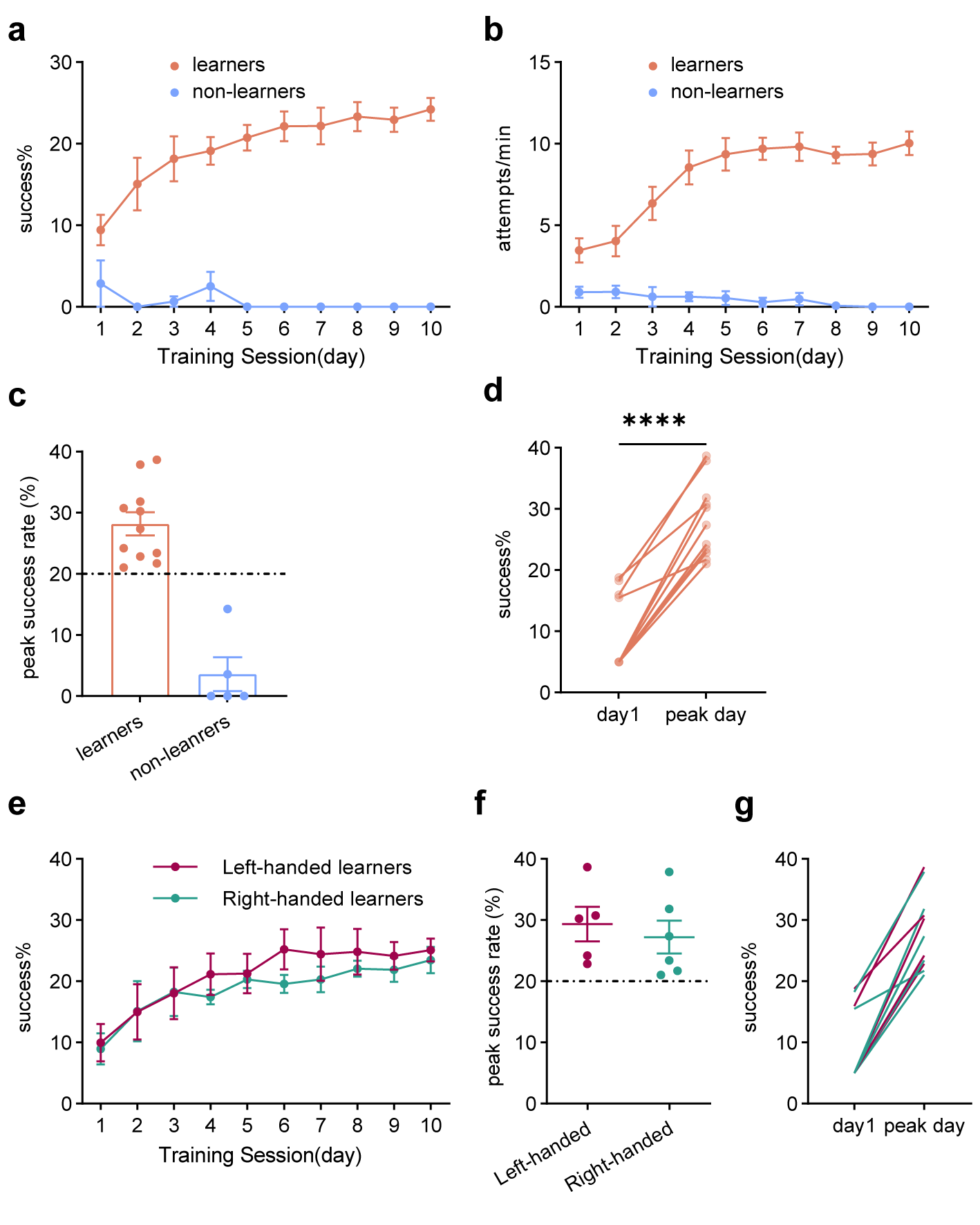
**

**Figure S1. Single pellet reaching task**

**a** Learners improve success rate over practice, non-learners maintain low success rate. Repeated-measured one-way ANOVA: in learners’ group, F (2.252, 22.52) = 9.440, p = 0.0008; in non-learners’ group, F (1.152, 4.610) = 1.168, p = 0.3455.

**b** Learners gradually increase their attempts during the first several days when success reach comes with food reward, then reach a plateau. In contrast, non-learners fail to get food pellets and lose motivation to reach.

**c** All learners achieve a peak success rate of over 20%, but non-learners fail to reach the line.

**d** Learners perform significantly better after training course. Day 1, 9.4%, peak day, 28.1%, n = 11 mice, paired two-tailed t test: t = 10.55, df = 10, p < 0.0001.

**e** Success rate curve of left-handed and right-handed mice. Repeated-measured one-way ANOVA: left-handed learners, n = 5 mice, F (2.471, 9.882) = 14.81, p = 0.0007; right-handed learners, n = 6 mice, F (3.326, 16.63) = 10.99, p = 0.0002.

**f** Comparison of peak success rate between left-handed and right-handed mice. n = 5 or 6 mice. Unpaired two-tailed t test: t = 0.5460, df = 9, p = 0.5983.

**g** Comparison of change in success rate between left-handed and right-handed mice. Paired two-tailed t test: left-handed, n = 5 mice, t = 8.585, df = 4, p = 0.0010, right-handed, n = 6 mice, t = 6.420, df = 5, p = 0.0014.

Data are shown as mean ± s.e.m. except for **d** and **g**. ns, no significance, p > 0.05, * p < 0.05, ** p < 0.01, *** p <0.001, **** p < 0.0001.


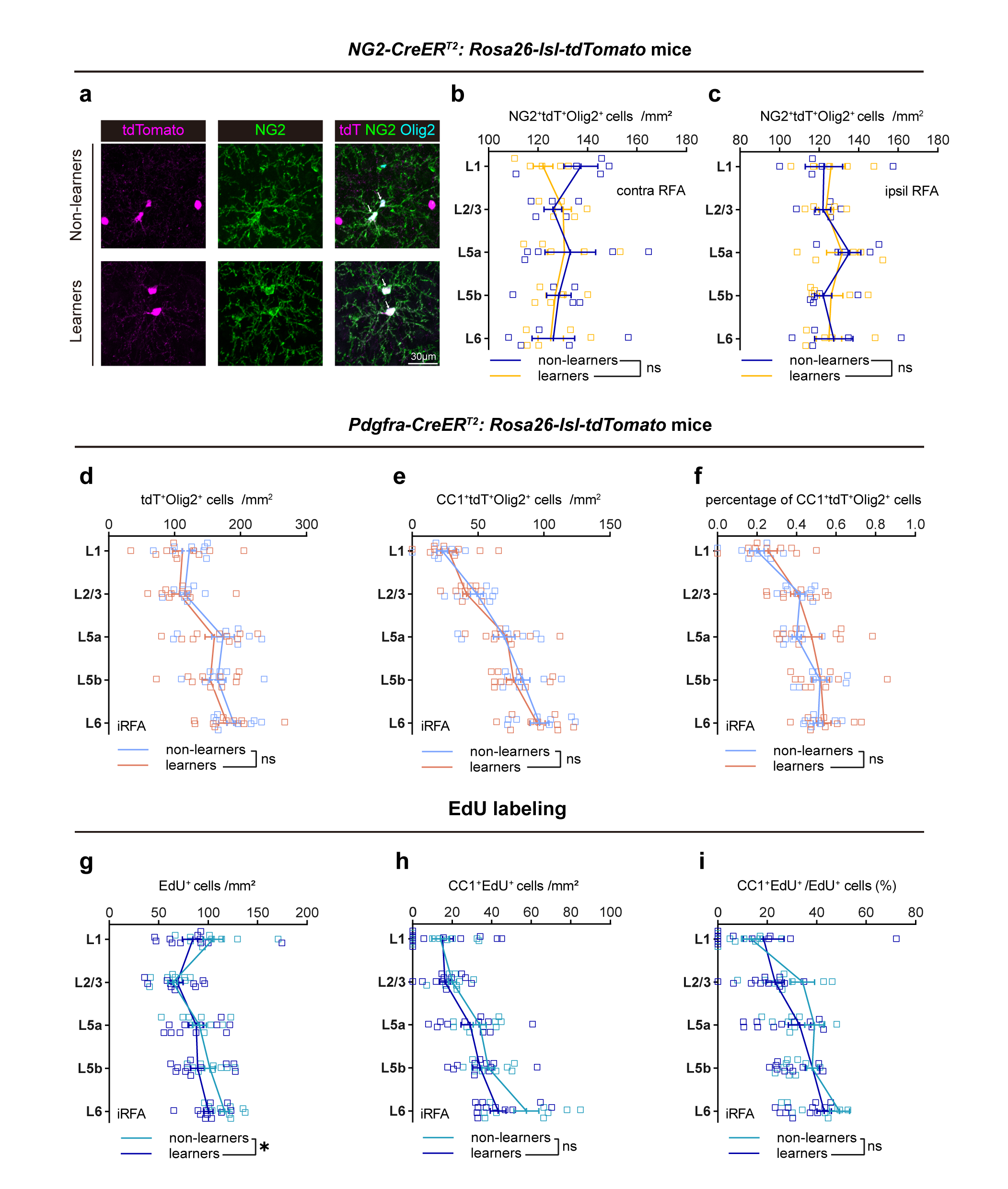


**Figure S2. Motor learning-induced OL changes in RFA**

**a** Representative image of immunostaining with tdTomato (magenta), Olig2 (cyan) and NG2 (green). Arrows denote tdT^+^NG2^+^Olig2^+^ cells.

**b** Cell number density of NG2^+^tdT^+^Olig2^+^ cells in cRFA of learners (n = 5 mice per layer) and non-learners (n = 5 mice per layer). Two-way ANOVA, non-learners vs. learners (training factor): F (1, 40) = 0.6617, p = 0.4208.

**c** Cell number density of NG2^+^tdT^+^Olig2^+^ cells in iRFA of learners (n = 5 mice per layer) and non-learners (n = 5 mice per layer). Two-way ANOVA, non-learners vs. learners (training factor): F (1, 40) = 0.0190, p = 0.8910.

**d-f** Cell number density of tdT^+^Olig2^+^**,** CC1^+^ tdT^+^Olig2^+^ and percentage of CC1^+^ tdT^+^Olig2^+^ cells in iRFA of learners (orange, n = 10 mice per layer) and non-learners (light blue, n = 8 mice per layer). **d,** non-learners vs. learners (training factor): F (1, 80) = 1.625, p = 0.2061. **e,** training factor: F (1, 80) = 0.2762, p = 0.6006; **f,** training factor: F (1, 80) = 1.575, p = 0.2132. Two-way ANOVA analysis.

**g-i** Cell number density of EdU^+^ **(g),** CC1^+^EdU^+^ **(h)** and percentage of CC1^+^EdU^+^ cells **(i)** in iRFA of learners (bule dash, n = 3 mice per layer) and non-learners (light blue dash, n = 4 mice per layer). **(g)** training factor: F (1, 90) = 4.230, p = 0.0426. **(h)** training factor: F (1, 90) = 3.719, p = 0.0570; **(i)** training factor: F (1, 90) = 1.611, p = 0.2077. Two-way ANOVA analysis.

**b-i**, data are shown as mean ± s.e.m., ns, no significance, p > 0.05, * p < 0.05.

**
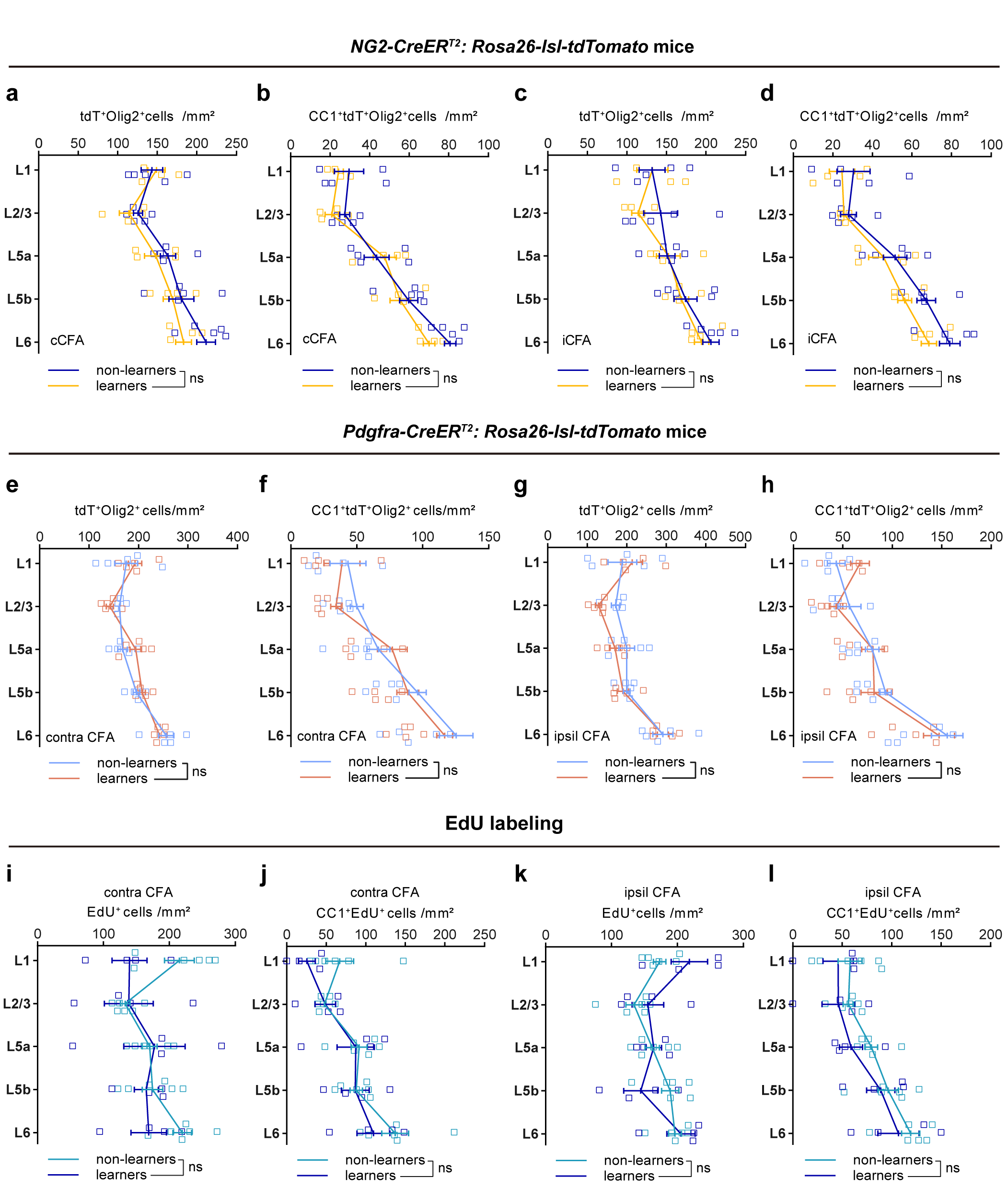
**

**Figure S3. Motor learning-induced OL changes in CFA**

**a-b** Cell number density of tdT^+^Olig2^+^**,** CC1^+^tdT^+^Olig2^+^ in cCFA and iCFA of learners (n = 4 mice per layer) and non-learners (n = 5 mice per layer). Two-way ANOVA: **a,** non-learners vs learners (training factor), F (1, 35) = 2.523, p = 0.1212; **b,** training factor, F (1, 35) = 2.192, p = 0.1477; **c,** training factor, F (1, 35) = 0.8844, p = 0.3534; **d,** training factor, F (1, 35) = 3.942, p = 0.0550.

**e-h** Cell number density of tdT^+^Olig2^+^**,** CC1^+^tdT^+^Olig2^+^ in cCFA and iCFA of learners (n = 4 mice per layer) and non-learners (n = 6 mice per layer). Two-way ANOVA: **(e)** non-learners vs learners (training factor), F (1, 40) = 1.403, p = 0.2431; **(f)** training factor, F (1, 40) = 0.07773, p = 0.7818; **(g)** training factor, F (1, 40) = 0.001020, p = 0.9747; **(h)** training factor, F (1, 40) = 0.004401, p = 0.9747.

**i-l** Cell number density of EdU^+^**,** CC1^+^EdU^+^ in cCFA and iCFA of learners (blue, n = 4 mice per layer) and non-learners (light blue, n = 6 mice per layer). Two-way ANOVA: **(i)** non-learners vs learners (training factor), F (1, 40) = 2.949, p = 0.0937; **(j)** training factor, F (1, 40) = 2.430, p = 0.1269; **(k)** training factor, F (1, 40) = 0.4011, p = 0.5301; **(l)** training factor, F (1, 40) = 2.689, p = 0.1089.

**a-l**, data are shown as mean ± s.e.m., ns, no significance, p > 0.05.


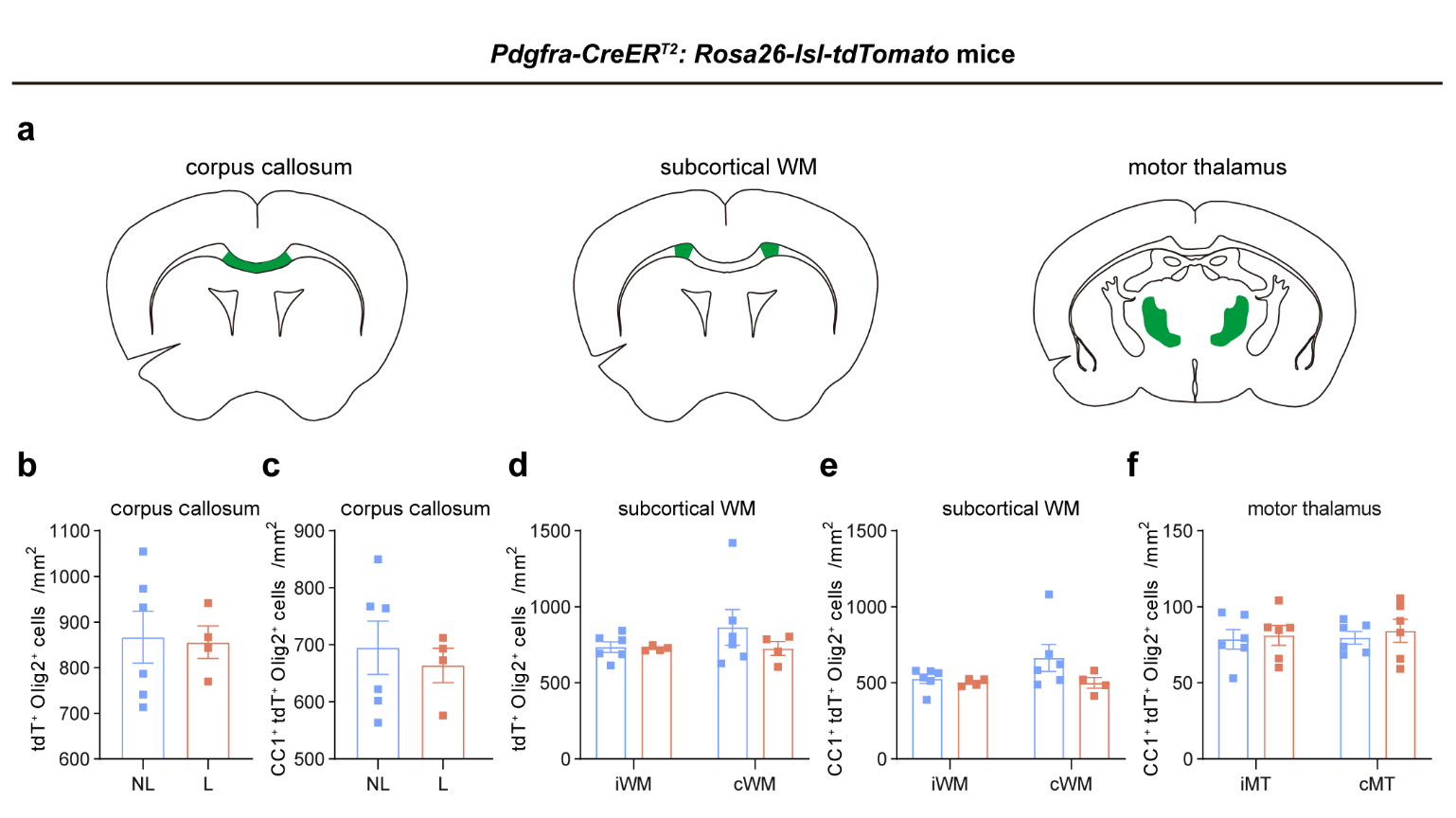


**Figure S4. Motor learning-induced OL changes in other motor-related regions**

**a** Schematic drawing of regions of interest, including corpus callosum, subcortical white matter (subcortical WM), and motor thalamus (MT).

**b-c** Quantification of tdT^+^Olig2^+^ cell density **(b)** and CC1^+^tdT^+^Olig2^+^ cell density **(c)** in corpus callosum between learners (L, n = 4 mice) and non-learners (NL, n = 6 mice). Data are shown as mean ± s.e.m. Unpaired two-tailed t test: **b**, t = 0.1460, df = 8, p = 0.8875, no significance, **c**, t = 0.4929, df = 8, p = 0.6354.

**d-e** Quantification of tdT^+^Olig2^+^ cell density **(d)** and CC1^+^tdT^+^Olig2^+^ cell density **(e)** in subcortical WM (ipsil- and contra-WM). Learners, orange, n = 4 mice, non-learners, blue, n = 6 mice, data are shown as mean ± s.e.m. **d**, in iWM groups, t = 0.2004, df = 8, p = 0.8462, in cWM groups, t = 0.9146, df = 8, p = 0.3872. **e**, in iWM groups, t = 0.6057, df = 8, p = 0.5615, in cWM groups, t = 1.432, df = 8, p = 0.1901. Unpaired two-tailed t test, no significance.

**f** Quantification of CC1^+^tdT^+^Olig2^+^ cell density in MT. Learners, orange, n = 6 mice, non-learners, blue, n = 6 mice, data are shown as mean ± s.e.m. Unpaired two-tailed t test: in iMT groups, t = 0.2905, df = 10, p = 0.7774, in cMT groups, t = 0.5089, df = 10, p = 0.6219, no significance.

**b-f**, data are shown as mean ± s.e.m.


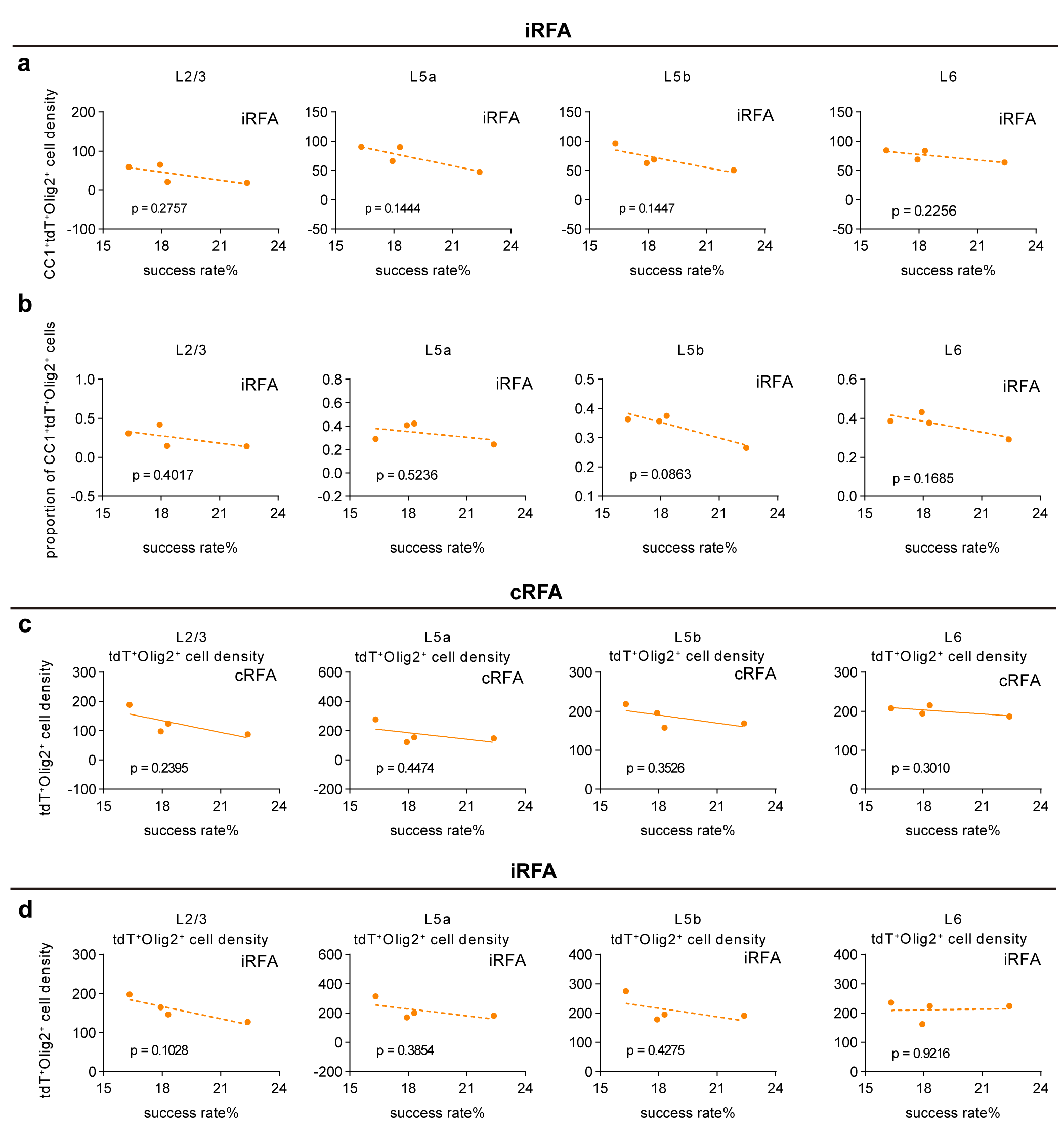


**Figure S5. Motor performance correlation with training-induced OL plasticity in NG2-tdT mice**

**a-b** Correlation between CC1^+^tdT^+^Olig2^+^ cell density or percentage of CC1^+^tdT^+^Olig2^+^ cells in iRFA with reaching performance (success rate) for individual NG2-tdT mice (n = 4 learners). Pearson correlation analysis, no significance, p > 0.05.

**c-d** Correlation between tdT^+^Olig2^+^ cell density with reaching performance (success rate) for individual NG2-tdT mice (n = 4 learners). Pearson correlation analysis, no significance, p > 0.05.


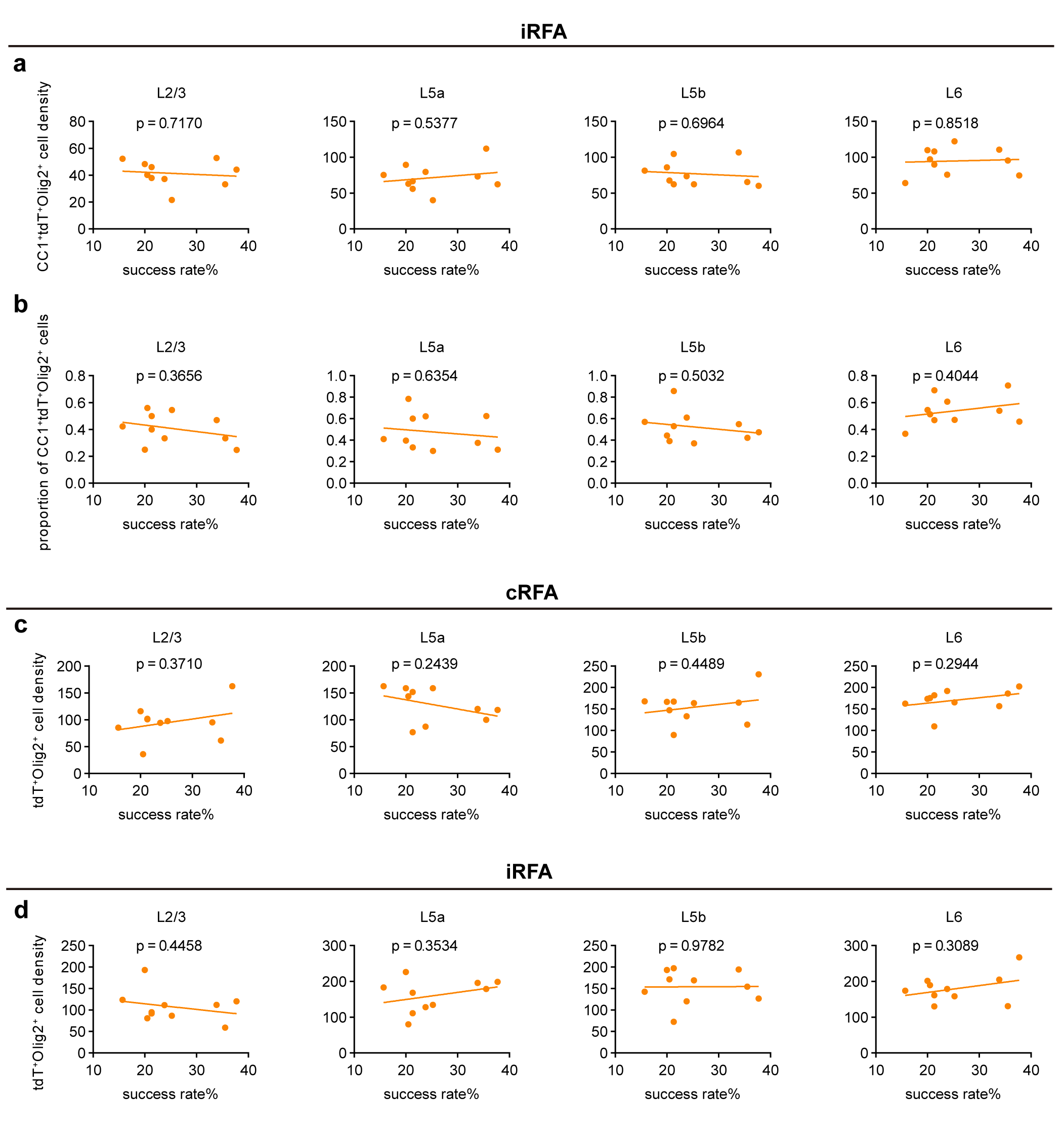
**Figure S6. Motor performance correlation with training-induced OL plasticity in P-tdT mice**

**a-b** Correlation between CC1^+^tdT^+^Olig2^+^ cell density or percentage of CC1^+^tdT^+^Olig2^+^ cells in iRFA with reaching performance (success rate) for individual P-tdT mice (n = 10 learners). Pearson correlation analysis, no significance, p > 0.05.

**c-d** Correlation between tdT^+^Olig2^+^ cell density with reaching performance (success rate) for individual P-tdT mice (n = 10 learners). Pearson correlation analysis, no significance, p > 0.05.


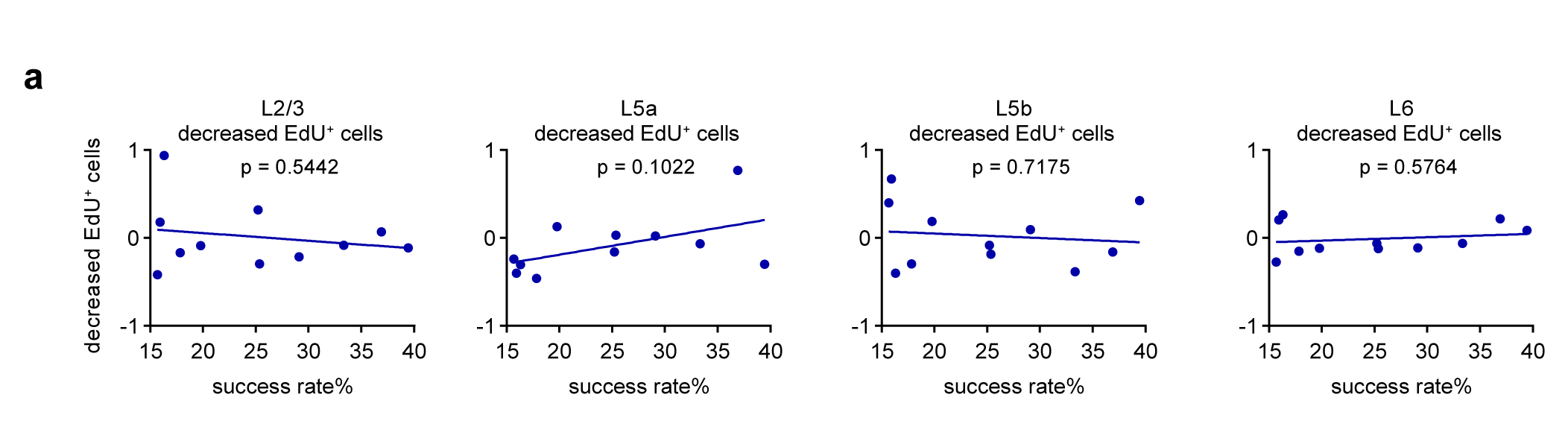
**Figure S7. Motor performance correlation with training-induced OL plasticity in EdU labeling mice**

**a** Correlation between decreased fractions of EdU^+^ cells relative to ipsil-RFA with reaching performance (success rate) for individual EdU labeling mice (n = 11 learners). Lines represent linear regression. Pearson correlation analysis, no significance, p > 0.05.

**
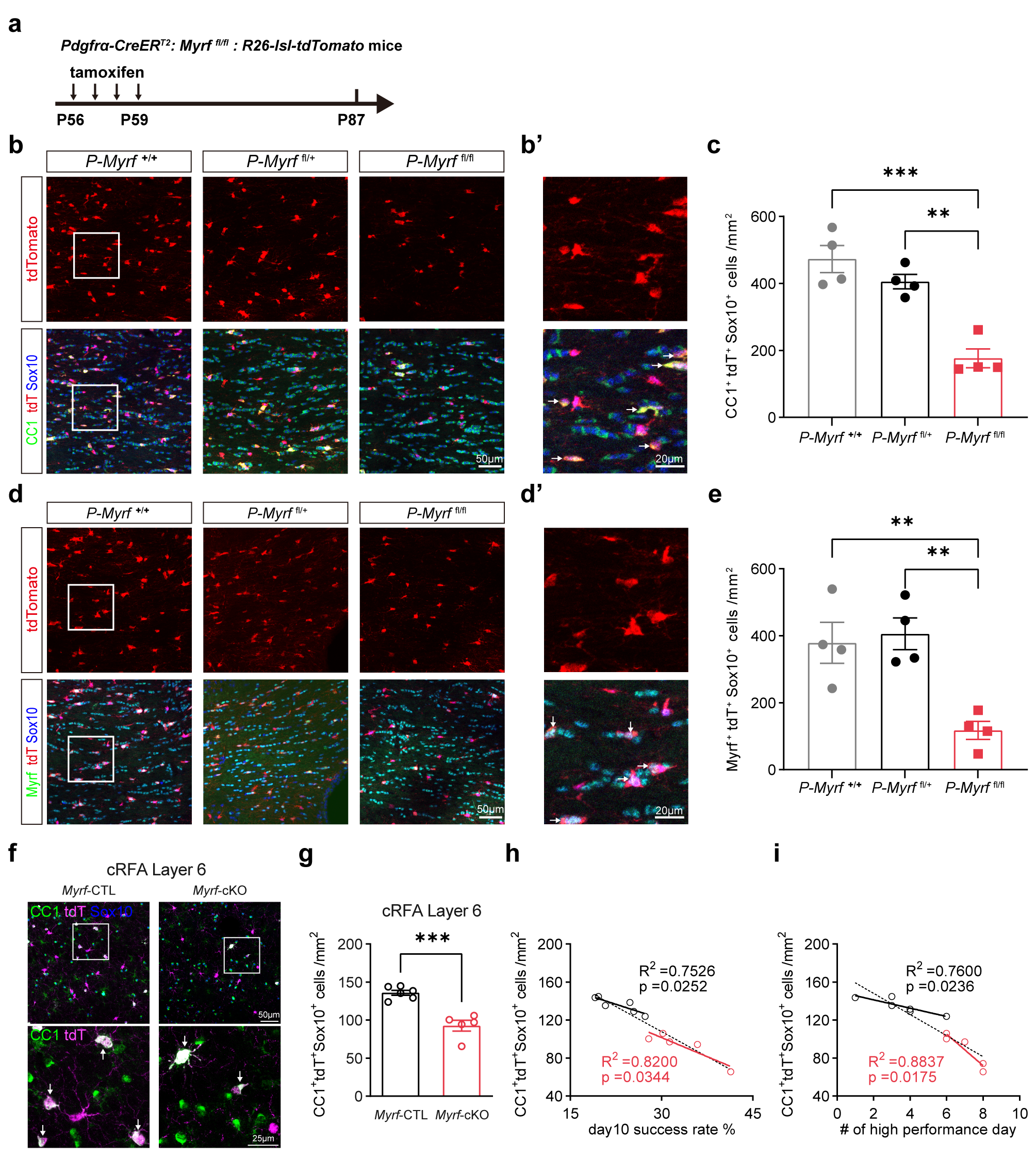
**

**Figure S8. *Myrf* conditional knockout regulates OL plasticity**

**a** Experimental paradigm for *Myrf* deletion and reaching task.

**b-b’** Representative staining image with tdTomato (red) and co-labeling with CC1 (green) and Sox10 (blue) in *Myrf*-cKO (*P-Myrf* ^fl/fl^) mice and control littermates including *P-Myrf* ^+/+^-*tdT* and *P-Myrf* ^fl/+^-*tdT* mice. **b’**, enlarged view of white square region in **b**.

**c** Comparison of CC1^+^tdT^+^Sox10^+^ cells across genotypes, *P-Myrf* ^+/+^ versus *P-Myrf* ^fl/fl^, adjusted p = 0.0002, *P-Myrf* ^fl/+^ versus *P-Myrf* ^fl/fl^, adjusted p = 0.0015, *P-Myrf* ^+/+^ versus *P-Myrf* ^fl/+^, adjusted p = 0.3219. One-way ANOVA analysis with Tukey's multiple comparisons test, n = 4 mice per group.

**d-d’** Representative staining image with tdTomato (red) and co-labeling with Myrf (green) and Sox10 (blue) in *P-Myrf* ^fl/fl^ mice and control littermates. **d’**, enlarged view of white square region in **d**.

**e** Comparison of Myrf^+^tdT^+^Sox10^+^ cells across genotypes, *P-Myrf* ^+/+^ versus *P-Myrf* ^fl/fl^, adjusted p = 0.0090, *P-Myrf* ^fl/+^ versus *P-Myrf* ^fl/fl^, adjusted p = 0.0050, *P-Myrf* ^+/+^ versus *P-Myrf* ^fl/+^, adjusted p = 0.9152. One-way ANOVA analysis with Tukey's multiple comparisons test, n = 4 mice per group.

**f** Representative image of tdTomato, CC1, and Sox10 in *Myrf*-CTL (*P-Myrf* ^fl/+^-*tdT*) learner mice (n = 6 mice) and *Myrf*-cKO (*P-Myrf* ^fl/fl^-*tdT*) learner mice (n = 5 mice).

**g** Comparison of CC1^+^tdT^+^Sox10^+^ cell density. Unpaired two-tailed t test, t = 5.868, df = 9, p = 0.0002.

**h** Correlation between CC1^+^tdT^+^Sox10^+^ cell density in L6 of cRFA with reaching performance (success rate) for individual mice. Pearson correlation analysis, *Myrf*-CTL, p = 0.0252, *Myrf*-cKO, p = 0.0344.

**i** Correlation between CC1^+^tdT^+^Sox10^+^ cell density in L6 of cRFA with reaching performance (number of high-performance days during 10-day session) for individual mice. Pearson correlation analysis, *Myrf*-CTL, p = 0.0236, *Myrf*-cKO, p = 0.0175.

* p < 0.05, ** p < 0.01, *** p <0.001. **c**, **e**, **g**, data are shown as mean ± s.e.m.

**
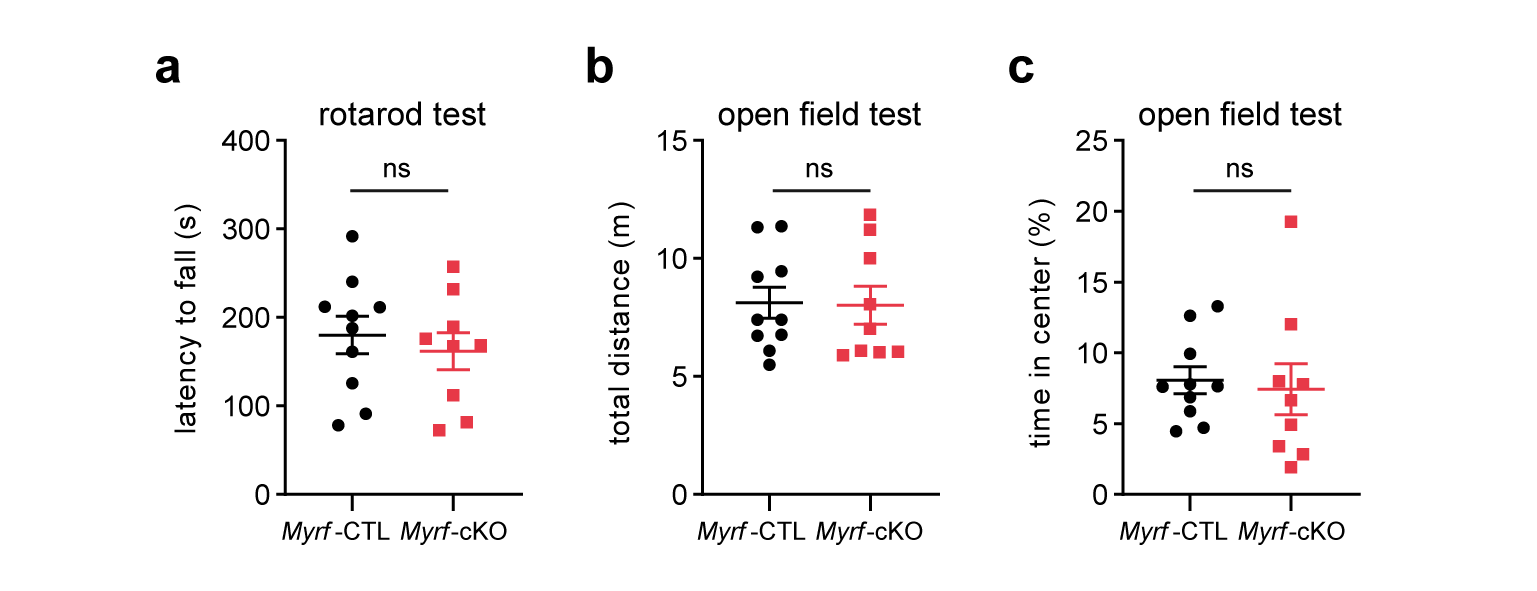
**

**Figure S9. Evaluation of general movement ability after *Myrf* conditional knockout.**

**a** Average latency to fall. n = 10 or 9 mice. Unpaired two-tailed t test, t = 0.6099, df = 17, p = 0.55.

**b** Total exploration distance. n = 10 or 9 mice. Unpaired two-tailed t test, t = 0.1002, df = 17, p = 0.9213.

**c** Proportion of exploring time in the center area. n = 10 or 9 mice. Unpaired two-tailed t test, t = 0.3297, df = 17, p = 0.7457.

Data are shown as mean ± s.e.m., ns, no significance, p > 0.05.


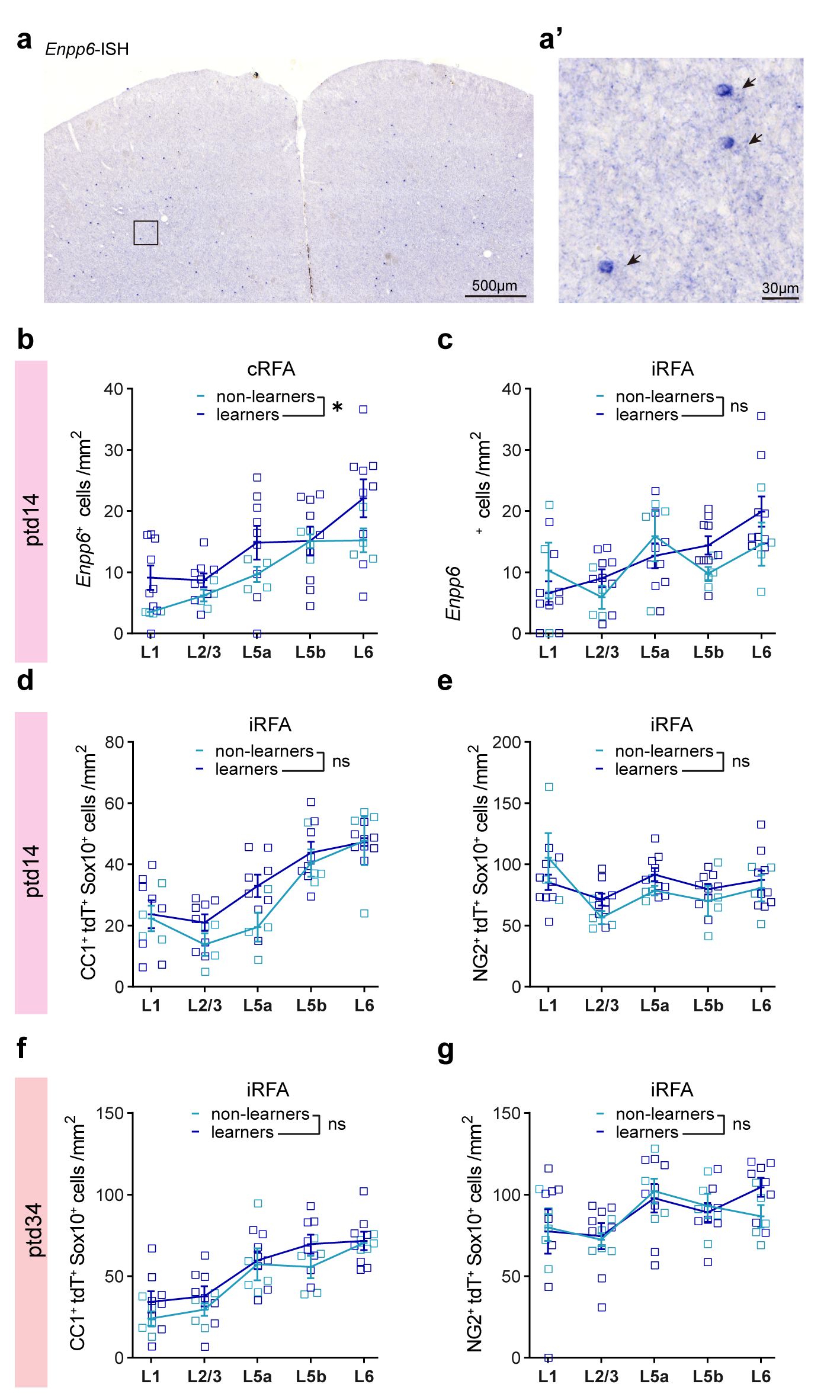


**Figure S10. Prolonged oligodendrocyte dynamics in rostral forelimb area after learning**

**a-a’** Representative image of *Enpp6* by in situ hybridization of RFA region. **a**: an overview of the upper brain slice. **a’**: enlarged view in black square, arrows denote *Enpp6*^+^ cells.

**b-c** Quantification of *Enpp6*^+^ cells number in cRFA **(b)** and iRFA **(c)** of learners (n = 9 mice per layer) and non-learners (n = 4 mice per layer) mice at ptd14. Two-way ANOVA: in cRFA, non-learners vs learners (training factor), F (1, 55) = 5.900, p = 0.0184, in iRFA, non-learners vs learners, F (1, 55) = 0.5586, p = 0.4580.

**d-e** Cell density of CC1^+^tdT^+^Sox10^+^ **(d)** and NG2^+^tdT^+^Sox10^+^ **(e)** in iRFA of learners (n = 8-9 mice per layer) and non-learners (n = 4 mice per layer) mice at ptd14. Two-way ANOVA: **d**, non-learners vs learners (training factor), F (1, 50) = 3.429, p = 0.0700, **e**, training factor, F (1, 55) = 0.8668, p = 0.3643.

**f-g** Cell density of CC1^+^tdT^+^Sox10^+^ **(f)** and NG2^+^tdT^+^Sox10^+^ **(g)** in iRFA of learners (n = 8 mice per layer) and non-learners (n = 5 mice per layer) mice at ptd34. Two-way ANOVA: **f**, non-learners vs learners (training factor), F (1, 55) = 3.192, p = 0.0795, **g**, training factor, F (1, 55) = 0.1098, p = 0.7416.

**b-g**, data are shown as mean ± s.e.m., ns, no significance, p > 0.05, * p < 0.05.


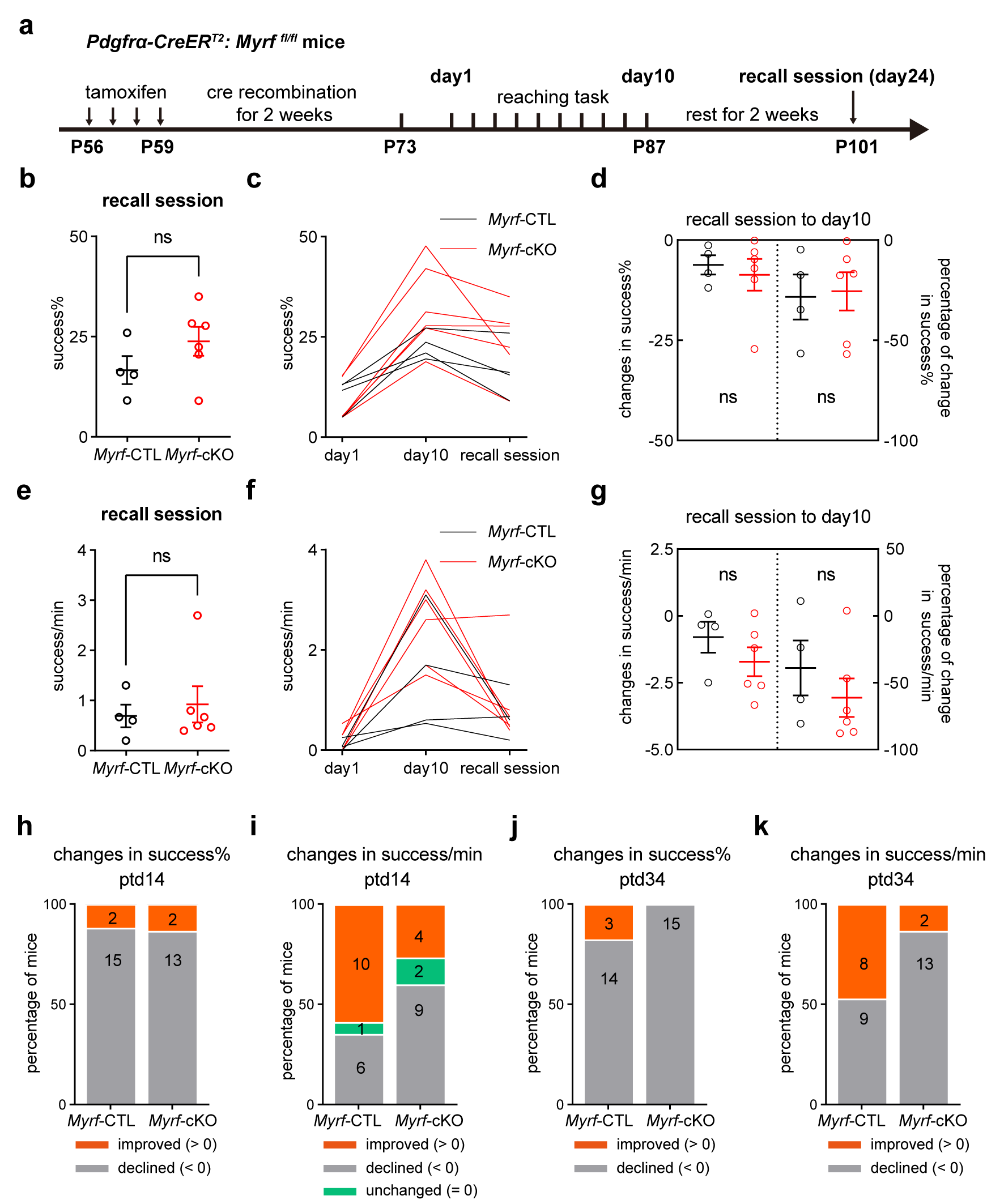


**Figure S11. Blocking oligodendrogenesis in *Myrf*-cKO mice before SPRT learning impairs motor skill maintenance**

**a** Experimental timeline to assess motor memory in *Myrf-*cKO mice prior to learning.

**b** Success rate of recall session in *Myrf*-CTL (n = 4 mice) and *Myrf*-cKO (n = 6 mice) groups. Data are presented as mean ± s.e.m. Unpaired two-tailed t test: t = 1.358, df = 8, p = 0.2116.

**c** Individual comparison of changes in success rate in *Myrf*-CTL (n = 4 mice) and *Myrf*-cKO (n = 6 mice) groups.

**d** Changes and percentages of change in success rate in recall session relative to day10 in *Myrf*-CTL (n = 4 mice) and *Myrf*-cKO (n = 6 mice) groups. Data are presented as mean ± s.e.m. Left, *Myrf*-CTL, -6.20% ± 2.39, *Myrf*-cKO, -8.64% ± 3.94. Right, *Myrf*-CTL, -28.36% ± 11.23, *Myrf*-cKO, -25.53% ± 9.51. Unpaired two-tailed t test: changes in success rate: t = 0.4633, df = 8, p = 0.6555; percentage of change in success rate: t = 0.1908, df = 8, p = 0.8535.

**e** Success trials per minute of recall session in *Myrf*-CTL (n = 4 mice) and *Myrf*-cKO (n = 6 mice) groups. Data are presented as mean ± s.e.m. Unpaired two-tailed t test: t = 0.4752, df = 8, p = 0.6474.

**f** Individual comparison of changes in success trials per minute in *Myrf*-CTL (n = 4 mice) and *Myrf*-cKO (n = 6 mice) groups.

**g** Changes and percentages of change in success trials per minute in recall session relative to day10 in *Myrf*-CTL (n = 4 mice) and *Myrf*-cKO (n = 6 mice) groups. Data are presented as mean ± s.e.m. Left, *Myrf*-CTL, -0.79 ± 0.58, *Myrf*-cKO, -1.71 ± 0.54. Right, *Myrf*-CTL, -38.89% ± 20.49, *Myrf*-cKO, -61.16% ± 14.38. Unpaired two-tailed t test: changes in success trials per minute: t = 1.131, df = 8, p = 0.2907; percentage of change in success trials per minute: t = 0.9203, df = 8, p = 0.3843.

ns, no significance, p > 0.05.

**h-k** Numbers and percentages of mice with improved (> 0), declined (< 0), or unchanged (= 0) motor performance after memory consolidation period.
